# Activation of AMPK inhibits cervical cancer growth by hyperacetylation of H3K9 through PCAF

**DOI:** 10.1186/s12964-024-01687-7

**Published:** 2024-06-03

**Authors:** Botao Pan, Can Liu, Jiyan Su, Chenglai Xia

**Affiliations:** 1 Foshan Women and Children Hospital, Foshan, 528000 China; 2 School of Pharmaceutical Sciences, Southern Medical University, Guangzhou, 515150 China

**Keywords:** AMP-activated protein kinase (AMPK), H3K9ac, Cervical cancer, PCAF, Histone acetylation

## Abstract

**Background:**

Dysregulation in histone acetylation, a significant epigenetic alteration closely associated with major pathologies including cancer, promotes tumorigenesis, inactivating tumor-suppressor genes and activating oncogenic pathways. AMP-activated protein kinase (AMPK) is a cellular energy sensor that regulates a multitude of biological processes. Although a number of studies have identified the mechanisms by which AMPK regulates cancer growth, the underlying epigenetic mechanisms remain unknown.

**Methods:**

The impact of metformin, an AMPK activator, on cervical cancer was evaluated through assessments of cell viability, tumor xenograft model, pan-acetylation analysis, and the role of the AMPK-PCAF-H3K9ac signaling pathway. Using label-free quantitative acetylproteomics and chromatin immunoprecipitation-sequencing (ChIP) technology, the activation of AMPK-induced H3K9 acetylation was further investigated.

**Results:**

In this study, we found that metformin, acting as an AMPK agonist, activates AMPK, thereby inhibiting the proliferation of cervical cancer both in vitro and in vivo. Mechanistically, AMPK activation induces H3K9 acetylation at epigenetic level, leading to chromatin remodeling in cervical cancer. This also enhances the binding of H3K9ac to the promoter regions of multiple tumor suppressor genes, thereby promoting their transcriptional activation. Furthermore, the absence of PCAF renders AMPK activation incapable of inducing H3K9 acetylation.

**Conclusions:**

In conclusion, our findings demonstrate that AMPK mediates the inhibition of cervical cancer growth through PCAF-dependent H3K9 acetylation. This discovery not only facilitates the clinical application of metformin but also underscores the essential role of PCAF in AMPK activation-induced H3K9 hyperacetylation.

**Supplementary Information:**

The online version contains supplementary material available at 10.1186/s12964-024-01687-7.

## Background

Cervical cancer is considered one of the most prevalent cancers among women, with approximately 300,000 fatalities and 600,000 new cases recorded annually on a global scale [1]. The identification of the causative pathogen, high-risk human papillomavirus (hrHPV), has revealed that persistent hrHPV infection is a primary cause of cervical cancer [2]. Predominantly, HPV types 16 and 18 are responsible for most cases, while a dozen other hrHPV types are linked to the remaining cases [3]. Although HPV infection is prevalent, the progression to cervical cancer necessitates further epigenetic and genetic alterations [4]. Recent research indicates that epigenetic anomalies are integral to the initiation and progression of cervical cancer [5]. These anomalies not only trigger viral oncogene activation post-HPV infection but are also crucial in the regulation of cellular differentiation and tumor advancement [6, 7].

Histone acetylation, a critical epigenetic mechanism, modulates transcriptional activity by maintaining the equilibrium between the opposing actions of histone deacetylases (HDACs) and histone acetyltransferases (HATs) [7]. This equilibrium is critical for the transcription and expression of numerous oncogenes, and its disruption by the dysregulated expression of HDACs and HATs can influence tumor progression. For instance, the overexpression of HDAC10 suppresses the expression of matrix metalloproteinase 2 and 9, thereby impeding the metastasis of cervical cancer [8]. Similarly, elevated levels of SIRT1 in HPV-infected cervical cancer cells sustain cell proliferation by repressing AIM2transcription [9]. Correlations have been observed between HPV 16 gene expression and histone acetylation levels on the HPV 16 genome [10]. Moreover, the acetyltransferase PCAF, acting as a co-activator of the tumor suppressor p53, is instrumental in tumor progression [11]. Consequently, the development of HAT and HDAC inhibitors as therapeutic targets to modulate histone acetylation levels is emerging as a promising strategy in oncology [12, 13]. These histone acetylation-based mechanisms also enhance our comprehension of the etiology and progression of cervical cancer.

AMP-activated protein kinase (AMPK), a heterotrimeric serine/threonine protein kinase, functions as a vital energy sensor and regulator. The activation of AMPK regulates various signaling networks that collectively mediate epigenetic transcriptional events, affirming AMPK’s role as a key player in epigenetic regulation [14]. While the modulation of AMPK activation holds promise for treating metabolic disorders, including type 2 diabetes [15], its association with epigenetic regulation suggests a significant role in tumorigenesis as well [16, 17]. Metformin, an agonist of AMPK, has garnered significant attention as a potential anti-cervical cancer therapeutic [18, 19]. Extensive research has demonstrated that metformin’s anti-tumor effects are mediated through epigenetic modifications [20–22]. Furthermore, metformin adjusts the activity of various epigenetic enzymes upon AMPK activation, consequently modifying lysine acetylation levels and impacting disease progression [22–24]. Although the specific functional implications of each histone acetylation modification remain elusive, aberrant patterns of histone acetylation alterations have been widely associated with cancer, affecting both the genome-wide landscape and specific genetic loci [25, 26]. Reports have indicated abnormal H3K9 acetylation modifications across a spectrum of human cancers [27–29]. This prompted our investigation into the potential of AMPK to induce H3K9 acetylation and the elucidation of its underlying regulatory mechanisms.

In this study, we observed that AMPK activation suppressed the in vitro and in vivo proliferation of cervical cancer. Mechanistically, this activation leads to epigenetic induction of H3K9 acetylation, in which promotes chromatin remodeling and enhances the binding of H3K9ac to the promoters of several tumor suppressor genes, thereby triggering their transcriptional activation. Furthermore, AMPK activation’s ability to hyperacetylate H3K9 is negated in the absence of PCAF. These discoveries not only substantiate the significance of the AMPK-PCAF-H3K9ac axis in cervical cancer progression but also offer fresh perspectives on epigenetic therapeutic approaches. Moreover, they reinforce the potential of metformin as a clinical candidate for curbing tumorigenesis.

## Materials and methods

### Cell culture, treatment, and transfection

HeLa (HPV18-positive, Cat. NO. CL-0101) and SiHa (HPV16-positive, Cat. NO. CL-0210) obtained from Procell (Wuhan, China), were cultured in DMEM supplemented with 1% penicillin-streptomycin (PS) (Sigma, MO, USA) and 10% FBS (Gibco, NY, USA) under standard conditions (37 °C, 5% CO_2_).

DMSO and metformin hydrochloride were provided by Sigma-Aldrich (MO, USA). Dorsomorphin and MHY1485 were obtained from MedChemExpress (Shanghai, China). Dorsomorphin and MHY1485 were prepared in DMSO solutions, while metformin was prepared as aqueous solutions for in vitro studies. Cell samples were collected after 48-hour treatment with various drugs (0–20 mM metformin, 10 µM dorsomorphin, 10 µM MHY1485, 10 µM dorsomorphin + 10 mM metformin, or 10 µM MHY1485 + 10 mM metformin) for subsequent Western blotting analysis.

The small interfering RNA (siRNA) PCAF (si-PCAF) and siRNA negative control (si-NC) were bought from Sangon Biotech (Shanghai, China). The sequences for si-PCAF were as follows: si-PCAF#1, 5`-CCU AAA CCG CAU CAA CUA UUG TT-3`, and si-PCAF#2, 5`-GCU GGG ACA AUU UCA UAC AAU TT-3`. si-PCAF or si-NC was transiently transfected into cervical cancer cells for 48 h using Lipofectamine 3000 (Invitrogen, NY, USA), after which drug treatment was administered for an additional 48 h before cell samples were collected for subsequent Western blotting analysis.

### Cell proliferation assays

Logarithmic growing SiHa or HeLa cells were seeded in 96-well plates at the optimal density of 2500 cells/well. After 24 h, various concentrations of metformin were added, and the cells were cultured for an additional 72 h. The anti-proliferative effect of metformin was assessed by adding CCK-8 reagent, followed by analysis using a microplate reader (BioTek, USA).

### Tumor xenografts

All protocols for the study of animals involved were reviewed and approved by the Experimental Animal Ethics Committee of Guangdong Pharmaceutical University (Guangdong Province, China). Twenty-four nude mice (female, BALB/c, and 4-week-old) were housed and bred in SPF-grade animal facility, maintained at temperatures ranging from 22–25℃, with humidity levels between 40 and 60%, and a 12 hou dark/light cycle. A xenograft was established by injecting SiHa cells (around 5 × 10^6^ cells/0.1 mL) suspended in serum-free media into the right forelimb near the axilla of the mice. When the tumor diameter reached the specified criteria (0.3–0.5 cm), the mice were numbered and randomly assigned into 4 groups (6/group) and administered treatments by gavage: Control (Saline), Cisplatin (CIS, 2 mg/kg), Metformin (MET, 5 mg/kg), and Metformin (MET, 50 mg/kg). The tumor volume (V) was calculated using the formula V = 0.5×A×B×B, where A is the longest and B is the shortest diameter of the mice’s subcutaneous tumors. Therefore, the tumor measurements were recorded every three days to calculate the tumor volume. After the treatment was completed, the sacrificed mice were promptly dissected to collect tumor xenografts for further studies.

### Western blot analysis

Briefly, whole protein and histone samples from cells or mouse tissues were obtained using a RIPA buffer (Beyotime; Shanghai, China) and a histone extraction kit (EpiGentek, Cat. NO. OP-0006, NY, USA), respectively. The western blot procedures followed the previous protocol [30]. The concentrations of the samples were quantified using a BCA assay (Beyotime; Shanghai, China), and then the proteins resolved by 8–15% SDS-PAGE were transferred onto a PVDF membrane (0.2 μm) (Millipore, MA, USA) and incubated sequentially with the primary antibody (0 °C, overnight) and the secondary antibody (room temperature, 2 h). Protein bands on the membranes were photographed using a ChampChemi chemiluminescence instrument (Sagacreation, Beijing, China) and an enhanced chemiluminescence solution (Thermo, MA, USA). ImageJ software wasused to analyze the immunoblot band intensities.

The antibodies used below and their corresponding secondary antibodies were bought from CST (Danvers, MA, USA), including anti-AMPKα (Cat#5832), anti-ACC (Cat#3676), anti-p70S6K (Cat#34,475), anti-mTOR (Cat#2983), anti-phosphorylation-AMPKα (Thr172) (Cat#2535), anti-phosphorylation-ACC (Ser79) (Cat#3661), anti-phosphorylation-p70S6K (Thr389) (Cat#9234), anti-phosphorylation-mTOR (Ser2448) (Cat#5536), anti-PCAF (Cat#3378), anti-SIRT2 (Cat#12,650), anti-PCNA (Cat#13,110), anti-β-actin (Cat#4970), anti-β-Tubulin (Cat#2146), and anti-GAPDH (Cat#5174). Other antibodies used in this study, anti-H3 (Cat#PTM-6613), anti-acetyllysine (Cat#PTM-105RM), anti-acetyl-H3K9 (Cat#PTM-112RM), and anti-acetyl-H3K56 (Cat#PTM-162), anti-acetyl-H3K122 (Cat#PTM-184), anti-acetyl-H2BK12 (Cat#PTM-108), anti-acetyl-H2BK20 (Cat#PTM-155), anti-acetyl-H2BK23 (Cat#PTM-174) were obtained from the PTM Biolabs Inc. (Hangzhou, China).

### qRT-PCR

A total RNA isolation kit (Cat. RE-03014, FOREGENE, Chengdu, China) was used to extract RNA from cell samples. cDNA was then synthesized from the RNA using PrimeScript RT Master Mix (TaKaRa, Japan), and the cDNA was then used for real-time PCR using SYBR green (TaKaRa, Japan). The relative expression level was calculated using the 2-ΔΔCt method with β-actin as the internal control. Primers for real-time PCR are listed below (F: forward; R: reverse): KAT2B (5’ -3’), CGAATCGCCGTGAAGAAAGC (F), CTTGCAGGCGGAGTACACT (R); SIRT2 (5’ -3’), ATCCACCGGCCTCTATGACAA (F), CGCATGAAGTAGTGACAGATGG (R).

### Protein extraction and digestion, TMT labeling, and acetyl peptide enrichment

Three pairs of independent replicate cell samples (10 mM metformin-treated SiHa cells and solvent-treated control SiHa cells) were collected. The experimental procedure was conducted following the protocol from the previous study [31]. After sonicating the samples (3 times, 0 °C) using a Scientz sonicator in lysis buffer and centrifuging to collect the supernatant (10 min, 12,000 g, 4 °C), the protein concentration was finally quantified using a BCA kit. Protein samples were treated with 5 mM DTT for reduction (30 min, 56 °C) and alkylated with 11 mM IAA (15 min, room temperature) in the dark, then diluted with 100 mM TEAB to achieve a urea concentration of less than 2 M. Next, the samples were treated with trypsin for digestion at 37 °C for 18 h.

Subsequently, the trypsin-digested peptide samples were processed according to the TMT kit protocol, following a series of operations that included desalting with a Phenomenex Strata X C18 SPE column, drying in vacuo, and reconstituted in 0.5 M TEAB. Then, the samples dissolved in NETN buffer were mixed with pre-washed anti-acetyllysine antibody-conjugated agarose beads (Cat. NO. PTM-104, PTM Bio, China) at 4 °C with moderate shaking, and incubated overnight to enrich for the modified peptides. Following this, the beads were sequentially washed with NETN buffer and then with H_2_O. Bound peptides from the beads were eluted with TFA (0.1%), and the eluted fractions were mixed and vacuum-dried.

### LC-MS/MS analysis

The experimental procedure was carried out according to the protocol of the previously study [31]. The resultant peptides were first desalted using C18 ZipTips (Millipore) before analysis by LC-MS on an EASY-nLC 1000 UPLC system, equipped with a homemade reversed-phase analytical column (15 cm length × 75 μm inner diameter), at a constant flow rate of 400 nL/min. Mobile phase A consisted of an aqueous solution with 0.1% formic acid and 2% acetonitrile, while mobile phase B consisted 0.1% formic acid and 90% acetonitrile. The liquid phase gradient settings were as follows: 10-25% solvent B, 0–24 min; 25-35% solvent B, 24–32 min; 35-80% solvent B, 32–36 min; 80% solvent B, 36–40 min. After separated by the UPLC system, the peptides were ionized with an NSI ion source and then analyzed by Q ExactiveTM Plus (Thermo) for mass spectrometry.

### Bioinformatic analysis

The MaxQuant search engine (v.1.5.2.8) was performed to evaluate the raw MS/MS data and compared it with the human UniProt database. Acetylated peptides having a fold-change of less than 1/1.5 or greater than 1.5 were regarded to have substantially differentially expression in this study (*P* < 0.05). The UniProt-GOA database (http://www.ebi.ac.uk/GOA/) and InterProScan software were employed to obtain GO annotated proteomes. The InterProScan in combination with the InterPro domain database (http://www.ebi.ac.uk/interpro/) also allows annotation of the functional descriptions of the identified protein domain. KEGG online software KAAS (http://www.genome.jp/kaas-bin/kaas_main) and the KEGG Mapper (http://www.kegg.jp/kegg/mapper.html) were used for KEGG enrichment analysis. The soft MoMo (http://meme-suite.org/tools/momo), motif-x algorithm was applied to analyze the motif characteristics of the modification sites.

The limma package in R software was utilized to identify the DEGs between the 24 normal cervical epithelial tissues and 33 primary tumors from the datasets GSE9750 in GEO (https://www.ncbi.nlm.nih.gov/geo). Subsequently, DEG volcano plots and scatter plots were obtained by the ggplot2 package in R language and the GraphPad 7.0 software. We employed R software and the ggplot2 package to analyze and visualize the correlation between the AMPK and PCAF. Initially, we downloaded RNAseq data from the TCGA-CESC (Cervical Squamous Cell Carcinoma and Endocervical Adenocarcinoma) project from the TCGA database. Subsequently, we performed data filtering by excluding normal samples, samples without clinical information, and duplicate data. Finally, we applied a log2 transformation (value + 1) to the data for subsequent analysis.

### Chromatin immunoprecipitation-sequencing

ChIP-seq was performed with the assistance of KangChen Biotechnology Co., Ltd. (Shanghai, China). Detailed experimental methodology can be found in previous studies [32, 33]. Briefly, SiHa cell samples from both control and 10 mM metformin-treated groups were analyzed using an H3K9ac antibody (Cat#ab32129, Abcam, USA). ChIP-Sequencing library preparation was performed according to Illumina’s protocol Preparing Samples for ChIP Sequencing of DNA. The libraries were denatured to generate single-stranded DNA molecules and sequenced on an Illumina NovaSeq 6000 following the NovaSeq 6000 S4 Reagent Kit (300 cycles) protocol. Similarly, data analysis can be performed following the methodology of published studies [32]. The detailed methods are available from the corresponding author upon reasonable request.

### Statistical analysis

In this study, data was analyzed using the GraphPad Prism 7.0 software. Data are presented as mean with standard deviation (SD). Statistical significance of differences between two groups and among multiple groups was assessed using an independent *t*-test and multivariate ANOVA, respectively. Unless otherwise noted, differences were considered to be statistically significant at *P* < 0.05.

## Results

### AMPK activation impedes cervical cancer tumor growth in vitro and in vivo

**Fig. 1:**
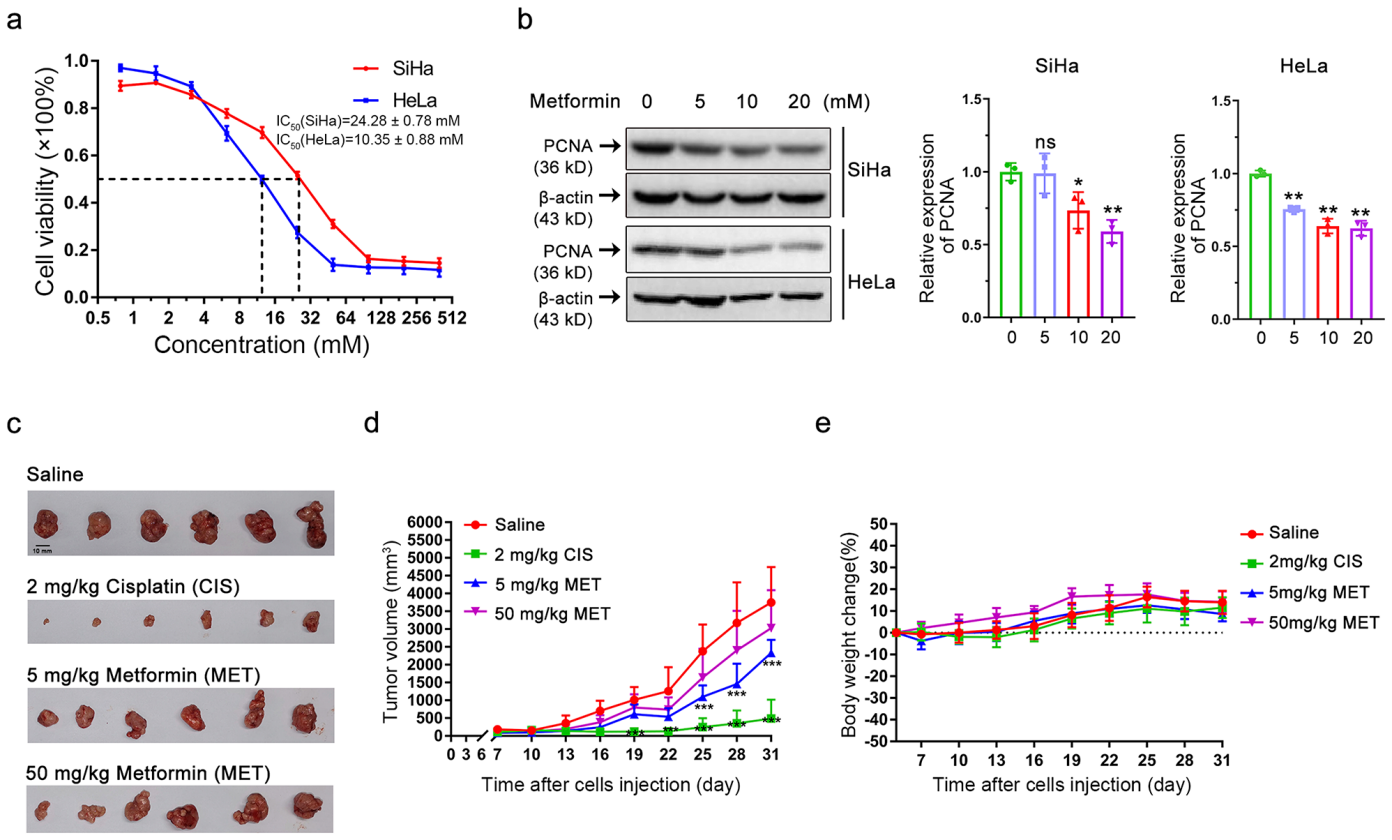
Metformin, an AMPK agonist, exerts cytotoxic and anti-tumor effects on cervical cancer. **(a)** The cell viability of HeLa and SiHa cells following metformin treatment was determined by CCK-8 assay. Data are means ± SD (*n* = 3). **(b)** Western blot analysis was performed to assess PCNA protein levels in cervical cancer cell lines HeLa and SiHa following metformin treatment (left). A statistical analysis of the expression of PCNA (right). Data represent means ± SD (*n* = 3). *P* values were calculated by one-way ANOVA. ^*^P < 0.05, ^**^P < 0.01, ns, not significant. **(c)** SiHa cell xenograft tumors from various groups, saline control group, 2 mg/kg cisplatin (CIS), 5 mg/kg metformin (MET), and 50 mg/kg metformin (MET). **(d)** Xenograft tumor growth curves of SiHa cells from different treatment groups. Tumor volumes formed in BALB/c nude mice were measured every 3 days after implantation of SiHa cells. Data represent means ± SD (*n* = 6). *P* values were calculated by multiple *t*-test. ^*^P < 0.05, ^**^P < 0.01, ^***^P < 0.001. **(e)** Body weight change (%) curves of mice in different treatment groups. The curves were plotted by collecting mouse weight data every 3 days after the implantation of SiHa cells (*n* = 6)

Data from clinical trials registered on ClinicalTrials.gov show that there are multiple studies using metformin in the treatment of a wide range of cancer types. In particular, some studies involving metformin in non-diabetic cancer patients have yielded promising results [34, 35]. Although recent studies by our group and others have found that metformin has significant anti-cervical cancer activity, the mechanisms behind this effect remain elusive [36–39]. To investigate this, we first used the CCK-8 assay to identify whether metformin induced anti-proliferative activities in different cervical cancer cells. Metformin dramatically inhibited cell proliferation in a concentration-dependent way, with IC_50_ results of 24.28 ± 0.78 mM and 10.35 ± 0.88 mM for SiHa and HeLa cells, respectively (Fig. 1a). PCNA [19], a molecular marker indicative of cell proliferation, was utilized to evaluate the growth status of SiHa and HeLa cells. As shown in Fig. 1b, the PCNA expression level dropped markedly in a concentration-dependent manner after a 48-hour treatment with metformin. These results suggest that metformin suppressed the proliferation of cervical cancer cells, implying a potential anti-tumor effect in vivo. To further define its effect on cervical cancer growth in vivo, we employed a SiHa cell xenograft model. The average tumor volume in the metformin and cisplatin groups was remarkably lower than the tumor volume in the saline group (Fig. 1c-d). Our results indicate that high doses of metformin (50 mg/kg) can reduce the growth rate of tumors, although this effect did not achieve statistical significance, which we suspect may be due to individual variability among the mice. Of note, no deaths or significant changes in body weight were observed in the metformin-intervention group throughout the experiment, suggesting that metformin is well-tolerated in mice with minimal side effects and low toxicity (Fig. 1e). Collectively, these findings highlight the potential antitumor efficacy of metformin both in vitro and in vivo.

**Fig. 2:**
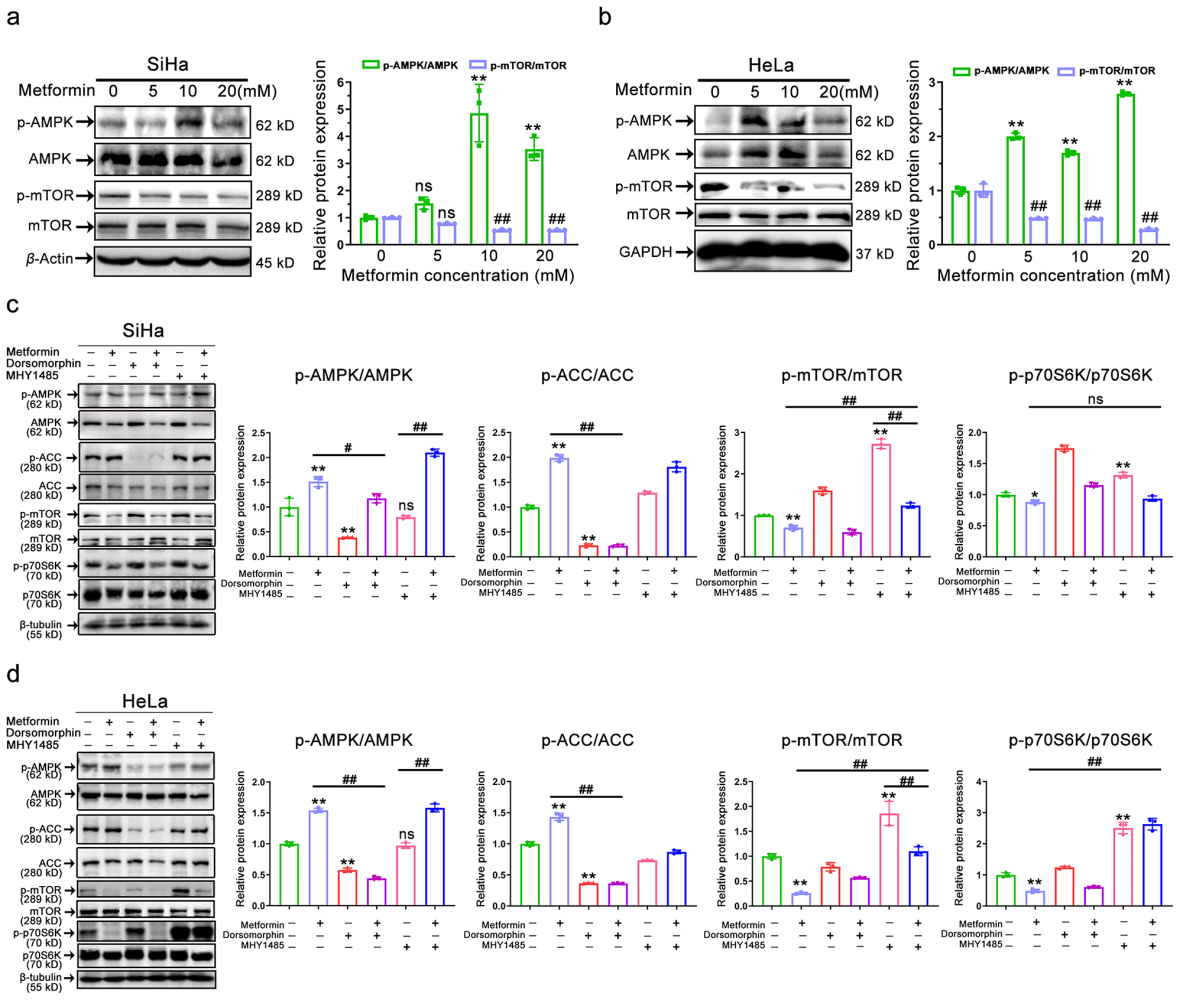
Effect of AMPK agonist metformin on the AMPK/mTOR pathway. (**a-b**) Representative immunostaining images (left) and their corresponding quantitative results (right) of the intensities of p-AMPK, AMPK, p-mTOR, and mTOR in metformin-treated (0 ~ 20 mM) SiHa (a) and HeLa (b) cells for 48 h. Data represent means ± SD (*n* = 3). *P* values were calculated using a one-way ANOVA test. ns, not significant; ^#^*P* or ^*^P < 0.05, ^##^*P* or ^**^P < 0.01 (vs. control group). **(c-d)** Representative immunostaining images (left) and their corresponding quantitative results (right) of the intensities of p-AMPK, AMPK, p-ACC, ACC, p-mTOR, mTOR, p-p70S6K, and p70S6K in SiHa (c) and HeLa (d) cells following treatment with dorsomorphin (10 µM), MHY1485 (10 µM), or metformin (10 mM). Data represent means ± SD (*n* = 3). *P* values were calculated by one-way ANOVA test. ns, not significant; ^#^*P* or ^*^P < 0.05, ^##^*P* or ^**^P < 0.01

Metformin, an AMPK agonist [40], led us to hypothesize that it might exert anti-tumor effects both in vitro and in vivo by activating AMPK. Furthermore, the modulation of mTOR by AMPK may be the most crucial anti-tumor effect of AMPK activation by metformin, as the inhibition of mTOR leads to downregulation of protein synthesis and cell proliferation [41]. To explore this, we examined the essential AMPK/mTOR axis using immunoblotting and found that metformin increased p-AMPK expression while decreasing p-mTOR expression (Fig. 2a-b). These results suggest that AMPK activation may inhibit the development of cervical cancer. Subsequently, we employed the small molecule tools dorsomorphin (an AMPK inhibitor) and MHY1485 (an mTOR activator) to interfere with the AMPK/mTOR pathway, aiming to further elucidate the role of metformin-activated AMPK in this context. As depicted in Fig. 2c-d, we assessed the phosphorylation levels of the AMPK, ACC (downstream targets of AMPK), mTOR, and p70S6K (downstream targets of mTOR) proteins that constitute this axis. Metformin treatment activated the AMPK/mTOR signaling pathway in two cervical cancer cell lines, whereas the AMPK inhibitor dorsomorphin or the mTOR activator MHY1485 had the opposite effect. Additionally, the activation of AMPK by metformin could be abrogated by the AMPK inhibitor dorsomorphin (p-AMPK/AMPK panel: bar 2 vs. 4, *P* < 0.05); similarly, the inhibition of mTOR by metformin was reversed by the mTOR agonist MHY1485 (p-mTOR/mTOR panel: bar 2 vs. 6, *P* < 0.05). Interestingly, the mTOR agonist MHY1485 did not significantly activate AMPK (p-AMPK/AMPK panel: bar 1 vs. 5, *P* > 0.05), but this was mitigated by metformin supplementation (p-AMPK/AMPK panel: bar 5 vs. 6, *P* < 0.05); although the mTOR agonist MHY1485 significantly activated mTOR (p-mTOR/mTOR panel: bar 1 vs. 5, *P* < 0.05), this was also mitigated by metformin supplementation (p-mTOR/mTOR panel: bar 5 vs. 6, *P* < 0.05). In conclusion, these data suggest that metformin activates the AMPK/mTOR axis, and that AMPK activation hinders cervical cancer tumor growth both in vitro and in vivo.

### AMPK activation alters the lysine acetylation pattern in cervical cancer

**Fig. 3:**
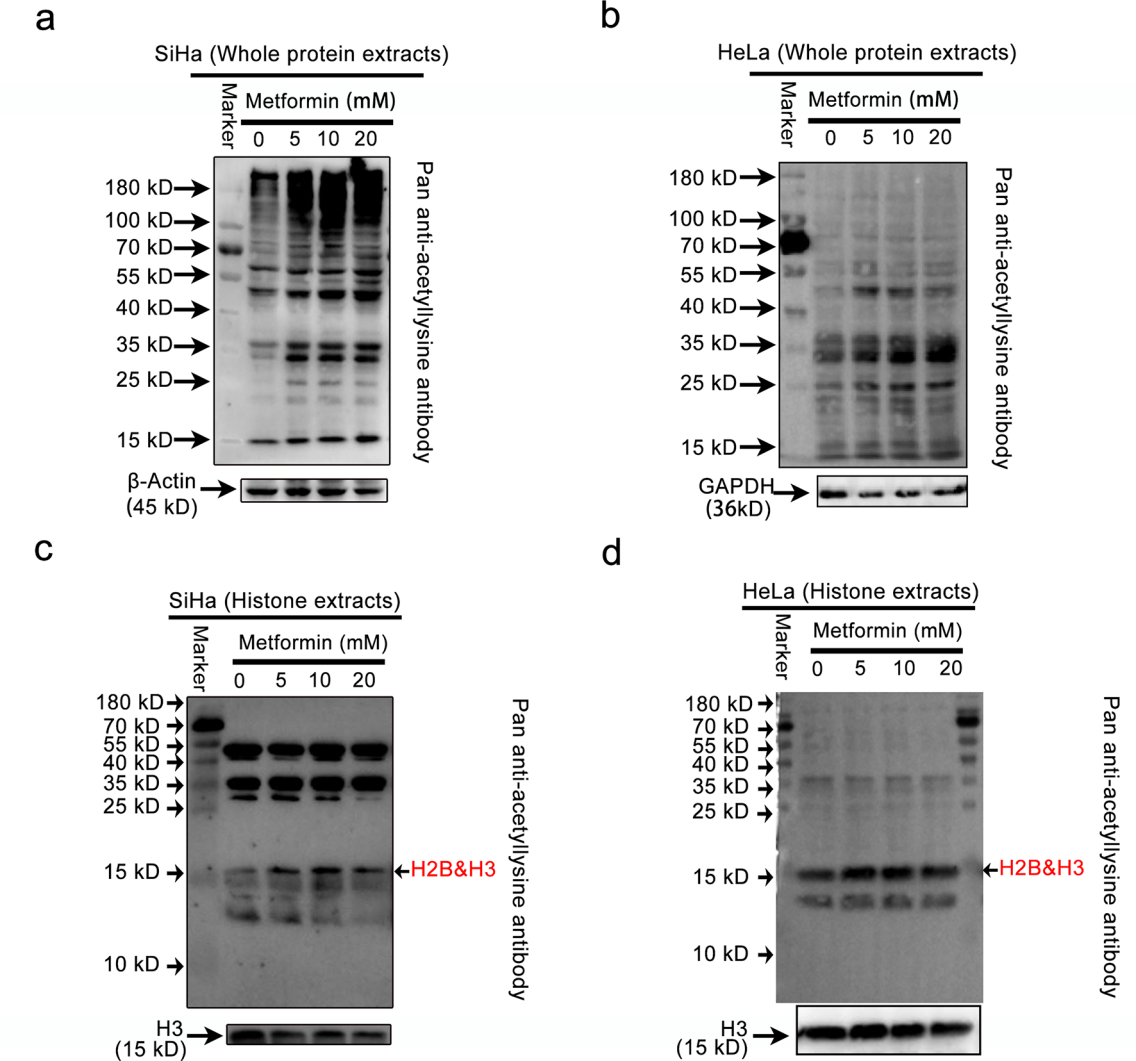
Profiling the global lysine acetylation of proteins through AMPK activation. **(a-b)** Representative Western blot images of the expression levels of acetylated proteins in whole protein extracts of SiHa (a) and HeLa (b) after metformin treatment. **(c-d)** Representative Western blot images of the expression levels of acetylated proteins in whole histone extracts of SiHa (c) and HeLa (d) after metformin treatment

As a potent AMPK activator, metformin has been demonstrated in recent years to induce histone and non-histone acetylation by activating AMPK [42]. Abnormalities in lysine acetylation have been implicated in cervical cancer tumorigenesis and could represent a prospective novel target for cancer treatment, especially the epigenetic alterations of histones, which can modulate chromatin architecture at promoter regions [13, 43]. However, there have been few published studies on the acetylation capacity of AMPK activation on lysine residues in cervical cancer. Given the preceding results showing that metformin activated AMPK in cervical cancer, we aimed to determine in vitro whether AMPK activation induces acetylation of whole protein and histone lysine residues. Cervical cancer cells treated with metformin were assessed for the acetylation level of lysine residues using a pan anti-acetylation immunoblot. Metformin efficiently altered the acetylation of lysine residues in whole protein extracts from SiHa cells, while only slightly affecting acetylation levels in HeLa cells (Fig. 3a-b). Of particular interest, metformin upregulated the acetylation levels of histone lysine residues (15 kD) in both cervical cancer cell lines (Fig. 3c-d). Thus, our results suggest that AMPK activation modifies the pattern of lysine acetylation in cervical cancer, particularly affecting histones. Encouraged by these findings, we hypothesized that AMPK activation would epigenetically upregulate histone acetylation modifications, increasing chromatin accessibility and thereby preventing the development of cervical cancer.

### Profiling the global lysine acetylation of proteins through AMPK activation

To further understand the comprehensive alteration of protein lysine acetylation in cervical cancer by AMPK activation, we performed a mass spectrometry-based quantitative acetylproteomic analysis. The overall workflow of the quantitative proteomics research for acetylation modification in this study is presented in Supplementary Fig. 1a. Our results identified more than 1000 proteins with a single lysine acetylation (Kac) site, approximately 400 proteins with two Kac sites, and over 200 proteins with three Kac sites (Supplementary Fig. 1b). Additionally, 5235 acetylation modification sites on 2069 proteins were discovered, with quantitative information available for 4804 sites on 1940 (Supplementary Fig. 1c, Additional file 1). Furthermore, 749 acetylated lysine sites corresponding to 509 proteins were significantly altered (fold change < 1/1.5 or > 1.5, and *P* < 0.05) after metformin treatment compared to the control, including 21 upregulated (acetylated) sites corresponding to 15 proteins, and 728 downregulated (nonacetylated) sites corresponding to 494 proteins (Supplementary Fig. 1d).

### Conserved motifs flanking the acetylation sites

Motifs of amino acid in the identified lysine-acetylated proteins (within 10 amino acids downstream and upstream of the acetylation site) were explored using motif-X to elucidate the characteristics of the amino acids surrounding the identified acetylation sites in metformin-treated protein samples. Supplementary Fig. 1e display the top 18 over-represented motifs. These motifs analyses revealed the presence of two groups of residues flanking the acetylated lysine. The first group included alanine (A), cysteine (C), lysine (K), and threonine (T) upstream of the Kac sites. The second group consisted of serine (S), histidine (H), tyrosine (Y), asparagine (N), proline (P), and tryptophan (W) downstream of the Kac sites, indicating a preference for residues K, Y, and T in protein lysine acetylation. The majority of the conserved residues were located at positions ± 1 or ± 2 from the Kac sites, based on the proximity of the residues to the acetylated lysine (Supplementary Fig. 1f). These motif models and residue preferences offer valuable insights for predicting the acetyl sites of unidentified acetyl-proteins.

### Functional annotation and subcellular localization of differentially acetylated proteins

Functional enrichment and subcellular localization studies were conducted to better understand the functions and characteristics of the identified differentially modified proteins. Firstly, gene ontology (GO) analysis and GO functional classification revealed that the identified Kac proteins were predominantly involved in single-organism processes, metabolic processes, cellular processes, developmental processes, and signaling (Supplementary Fig. 2a-c). Membrane, organelle, membrane-enclosed lumen, and extracellular regions were the main areas where the Kac proteins were distributed in cells, while catalytic activity and binding were the main functions of the identified Kac proteins (Supplementary Fig. 2d).

Next, the KEGG results showed that Kac proteins were involved in “antigen processing and presentation (hsa04612)”, “cholesterol metabolism (hsa04979)”, “non-homologous end-joining (hsa03450)”, and “one carbon pool by folate (hsa00670)”, based on statistically significant pathways (*P* < 0.05) (Supplementary Fig. 2e). Upregulated Kac proteins were notably enriched in pathways such as “systemic lupus erythematosus (hsa05322)”, “alcoholism (hsa05034)”, and “carbon metabolism (hsa01200)” (Supplementary Fig. 2f), while downregulated Kac proteins were significantly enriched in “cholesterol metabolism (hsa04979)”, “antigen processing and presentation (hsa04612)”, “non-homologous end-joining (hsa03450)”, and “protein processing in endoplasmic reticulum (hsa04141)” (Supplementary Fig. 2g).

In the subcellular localization classification, 41.09% of downregulated Kac proteins were localized in the cytoplasm, 30.16% in the nucleus, 10.53% in the mitochondria, 5.87% in extracellular areas, 4.05% in the plasma membrane, and 2.63% in other cellular regions (Supplementary Fig. 2h). For the upregulated Kac protein localization, 73.33% were in the nucleus, 20% were in the cytoplasm, and 6.67% were in the mitochondria (Supplementary Fig. 2i). These findings imply that lysine acetylation of proteins is present in various cellular processes and may be one of the pivotal regulatory mechanisms by which AMPK activation inhibits cervical cancer proliferation.

### AMPK activation induces H3K9 hyperacetylation in vitro

**Fig. 4:**
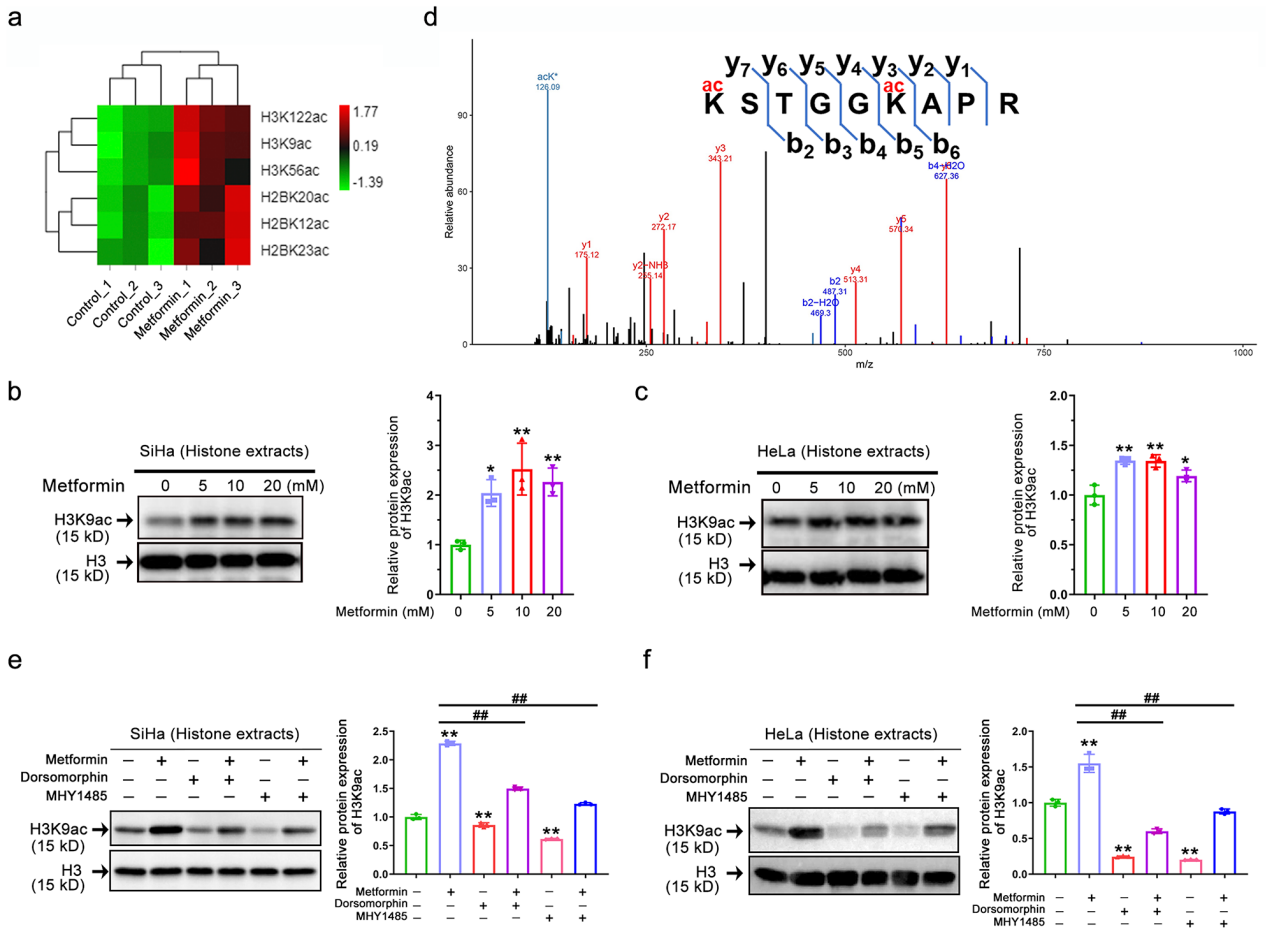
AMPK activation induces H3K9 hyperacetylation in vitro. **(a)** Heatmap analysis of metformin-altered acetylation levels at different lysine sites on histone H3 and H2B in cervical cancer cells based on quantitative acetylproteomic results (*n* = 3). **(b-c)** Representative immunostaining images (left) and quantitative results (right) of the H3K9ac intensity in whole histone extracts of SiHa (b) and HeLa (c) after metformin (0 ~ 20 mM) treatment. Data represent means ± SD (*n* = 3). *P* values were calculated by one-way ANOVA. ^*^P < 0.05, and ^**^P < 0.01. **(d)** Identification by LC-MS/MS of H3 4–12 peptides carrying acetylation (ac) at K9. **(e-f)** Representative immunostaining images (left) and quantitative results (right) of the H3K9ac intensity in SiHa (e) and HeLa (f) after dorsomorphin (10 µM) or (and) MHY1485 (10 µM) or (and) metformin (10 mM) treatment. Data represent means ± SD (*n* = 3). *P* values were calculated by one-way ANOVA. ^#^*P* or ^*^P < 0.05, ^##^*P* or ^**^P < 0.01

Hyperacetylation may be attributed to the expression of proto-oncogenes, whereas hypoacetylation is implicated in the silencing of tumor suppressor genes [26]. Acetylproteomic results have shown that histones were acetylated in SiHa cells following metformin therapy, including three lysine residues in both H3 (P84243) and H2B (Q99879). The acetylation-modified sites of H2B were K12, K20, and K23 residues, and those of H3 were K9, K56, and K122 residues, as identified by LC-MS/MS (Fig. 4a). Recent studies have shown that histone acetylation marks on K9 (H3K9ac) of H3 are related to the activation of gene expression and tumor progression; however, much remains unknown about their role in cervical cancer [29]. Given this, we next explored the role of acetylation at this site in cervical cancer after AMPK activation. As shown in Fig. 4b-c, after metformin treatment of SiHa and HeLa cells, we observed that AMPK activation strongly induced hyperacetylation of histone H3 at the Lys9 residue. To further validate the reliability of the acetylproteomics results, we performed Western blot validation for the acetylation of other lysine residues identified on histone H3/H2B (H3K122, H3K56ac, H2BK12, H2BK20, and H2BK23). The results were consistent with the mass spectrometry results, indicating that the acetylproteomics results are reliable (Supplementary Fig. 3). In addition, the MS/MS spectra results identified K9ac in H3 (Fig. 4d). Taken together, these results indicated that AMPK activation by metformin can hyperacetylate multiple H3/H2B lysine residues, but the regulatory mechanism behind this requires further investigation. Subsequently, we interfered with the AMPK/mTOR pathway using the small molecule tools dorsomorphin (AMPK inhibitor) and MHY1485 (mTOR activator) to further clarify the role of the AMPK/mTOR axis in the acetylation of H3K9. As shown in Fig. 4e-f, the level of H3K9ac was most strikingly upregulated during metformin treatment, while treatment with dorsomorphin (AMPK inhibitor) or MHY1485 (mTOR activator) significantly reduced this, suggesting that the AMPK/mTOR axis regulates H3K9ac. This conclusion is further corroborated by the fact that either dorsomorphin (AMPK inhibitor) or MHY1485 (mTOR activator) prevented the hyperacetylation of H3K9 induced by metformin.

### PCAF is essential for AMPK-activation-induced H3K9 hyperacetylation

**Fig. 5:**
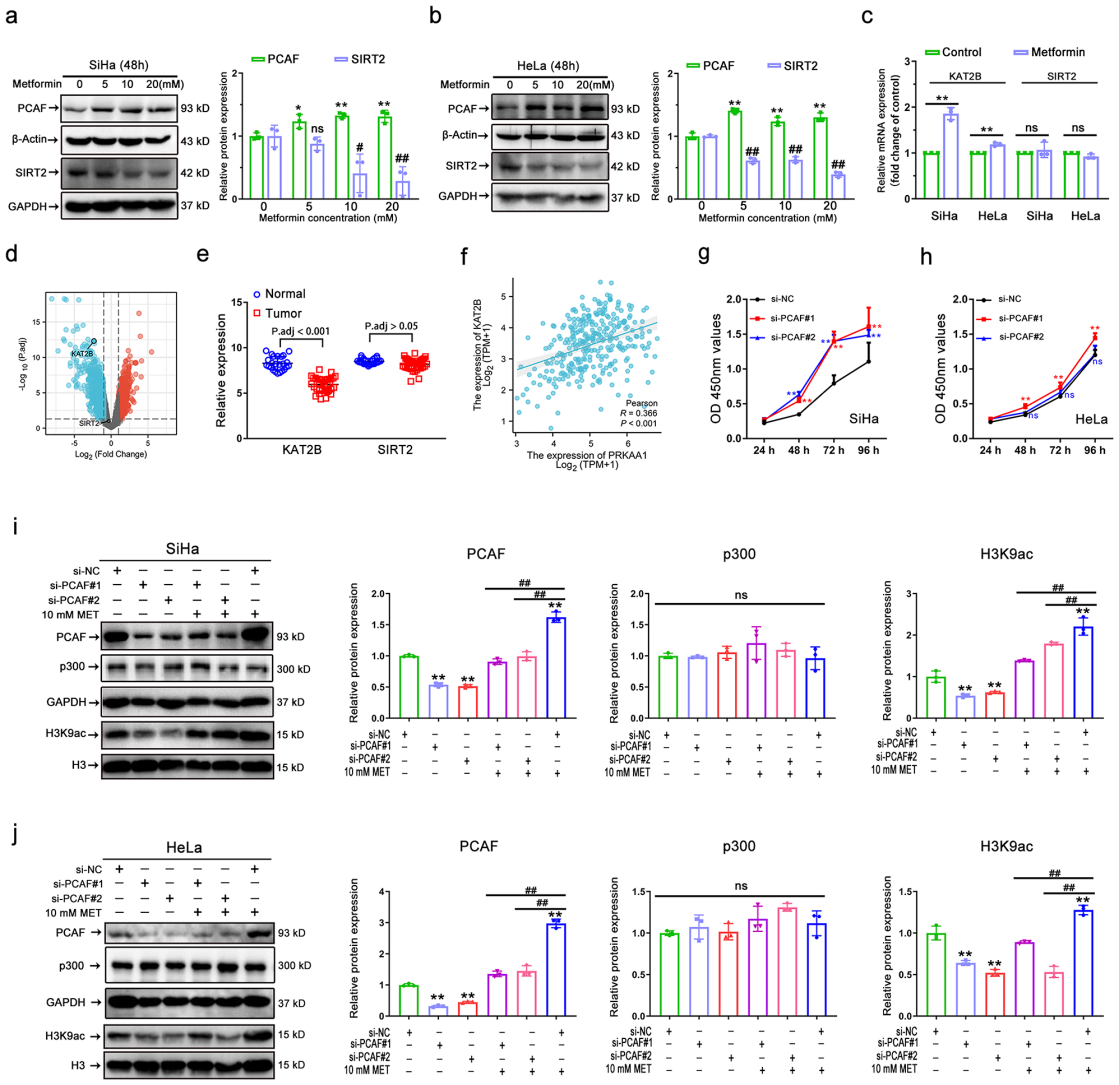
PCAF is essential for AMPK-activation-induced H3K9 hyperacetylation. **(a-b)** Representative immunostaining images (left) and quantitative results (right) of the PCAF and SIRT2 intensity in metformin (0 ~ 20 mM) treated SiHa (a) and HeLa (b) cells. **(c)** Relative mRNA expression levels of KAT2B, and SIRT2 in SiHa and HeLa after 10 mM metformin treatment (*n* = 3). **(d)** Volcano plots of significantly differentially expressed genes identified in the GEO database between 33 primary cervical cancer tissues and 24 normal cervical epithelial tissues. **(e)** Dot plots of the relative expression of KAT2B and SIRT2 genes between 33 primary cervical cancer tissues and 24 normal cervical epithelial tissues. **(f)** Correlation Analysis of AMPK (encoded by PRKAA1) and PCAF (encoded by KAT2B) Expression in the TCGA-CESC Dataset. **(g-h)** Cell growth curves under different treatments of si-NC, si-PCAF#1, and si-PCAF#2 in SiHa **(g)** and HeLa **(h)** cells (*n* = 3). (**i-j**) Representative immunostaining images (left) and quantitative results (right) of the PCAF, p300, and H3K9ac intensity in SiHa (i) and HeLa (j) cells after si-PCAF or (and) metformin (10 mM) treatment. Data are means ± SD. The two-tailed Student’s *t*-test (c) and one-way ANOVA (a/b/i/j) were used to performed comparison between two groups and more groups, respectively. For g/h, *P* values were calculated by multiple *t*-test. ns, not significant; ^#^*P* or ^*^P < 0.05; ^##^*P* or ^**^P < 0.01

It is well known that acetylation and deacetylation on specific lysine residues of histones can be catalyzed by HATs and HDACs, respectively, thereby epigenetically regulating global gene transcription and metabolism in tumors [44]. Recent studies have shown that SIRT2 mediates deacetylation of H3K9 [45], and the histone acetyltransferase KAT2B (also known as PCAF) has been identified as the key enzyme responsible for H3K9ac deposition [46]. To explore the potential mechanism by which AMPK activation upregulates H3K9ac, we performed western blot analysis on the H3K9ac histone modifiers (PCAF and SIRT2), which play a regulatory role in the histone acetylation mechanism. Among these histone-modifying factors, activation of AMPK by metformin significantly increased PCAF expression while decreasing SIRT2 expression (Fig. 5a-b). The differential expression patterns of PCAF and SIRT2 in AMPK-activated cervical cancer cells suggest that acetylation of H3K9 in cervical cancer may be dynamically regulated by acetyltransferases and deacetylases. In addition, we investigated whether metformin treatment altered the RNA expression levels of PCAF and SIRT2 genes. Metformin significantly increased the expression level of the PCAF gene (KAT2B) without affecting the expression level of the SIRT2 gene (Fig. 5c), suggesting that the AMPK activation-mediated H3K9ac upregulation may be associated with PCAF. To further evaluate the functions of PCAF and SIRT2 in cervical cancer, their transcriptional expression patterns were extracted from the GEO database in 33 primary tumors and 24 normal cervical epithelial tissues. PCAF gene mRNA levels were substantially downregulated in tumor tissue relative to normal cervical epithelial tissue, whereas SIRT2 levels were not significantly different, leading us to hypothesize that SIRT2 may not be a critical regulatory factor in this context (Fig. 5d-e). We have additionally performed a correlation analysis for AMPK (encoded by PRKAA1) and PCAF (encoded by KAT2B) utilizing data from the TCGA database (Fig. 5f). The findings reveal a statistically significant positive correlation between the expression levels of these two genes.

Furthermore, previous research has demonstrated that patients with elevated PCAF expression have an improved prognosis, and PCAF may be a novel anti-oncogene in cervical cancer [47]. However, whether PCAF is related to cervical cancer proliferation remains unknown. Thus, before demonstrating that AMPK activation can mediate PCAF upregulation to induce H3K9ac hyperacetylation and thus inhibit cervical cancer growth, it is necessary to determine whether PCAF inhibits cervical cancer proliferation. We utilized si-RNA to establish SiHa and HeLa cell lines with silenced PCAF to assess their impact on the proliferation of cervical cancer using the CCK8 assay. Concurrently, we employed si-NC as a negative control to ensure that the observed effects were due to the specific interaction between siRNA and the target gene, rather than nonspecific factors such as the transfection process or cellular responses. As illustrated in Fig. 5g-h, compared to the si-NC group, the silencing of PCAF enhanced the cell viability of both SiHa and HeLa cell lines. This suggests that PCAF functions as a tumor suppressor gene in cervical cancer.

Considering the role of PCAF as an acetyltransferase and its function in cancer, we next investigated whether metformin-mediated H3K9ac upregulation occurs via PCAF. Firstly, our results showed that global levels of H3K9ac were substantially reduced following siRNA-mediated silencing of PCAF expression in cervical cancer cell lines, indicating that impaired PCAF function may impact the capacity to acetylate H3K9 in cervical cancer (Fig. 5i-j). Then, we examined whether metformin supplementation could restore H3K9ac levels that were decreased by si-PCAF treatment. Even with metformin supplementation, the acetylation level of H3K9 could not be restored due to PCAF’s impaired acetylation function. Additionally, we investigated p300, another essential HAT. Our results showed that neither PCAF silencing nor metformin supplementation altered the expression level of p300, providing further evidence that PCAF is crucial for metformin-mediated hyperacetylation of H3K9. In conclusion, our results suggest that PCAF deficiency abolishes the metformin-induced H3K9 hyperacetylation effect, emphasizing the importance of PCAF in AMPK-activation-induced H3K9ac.

### AMPK/mTOR axis participation in the regulation of PCAF acetylation activity

**Fig. 6:**
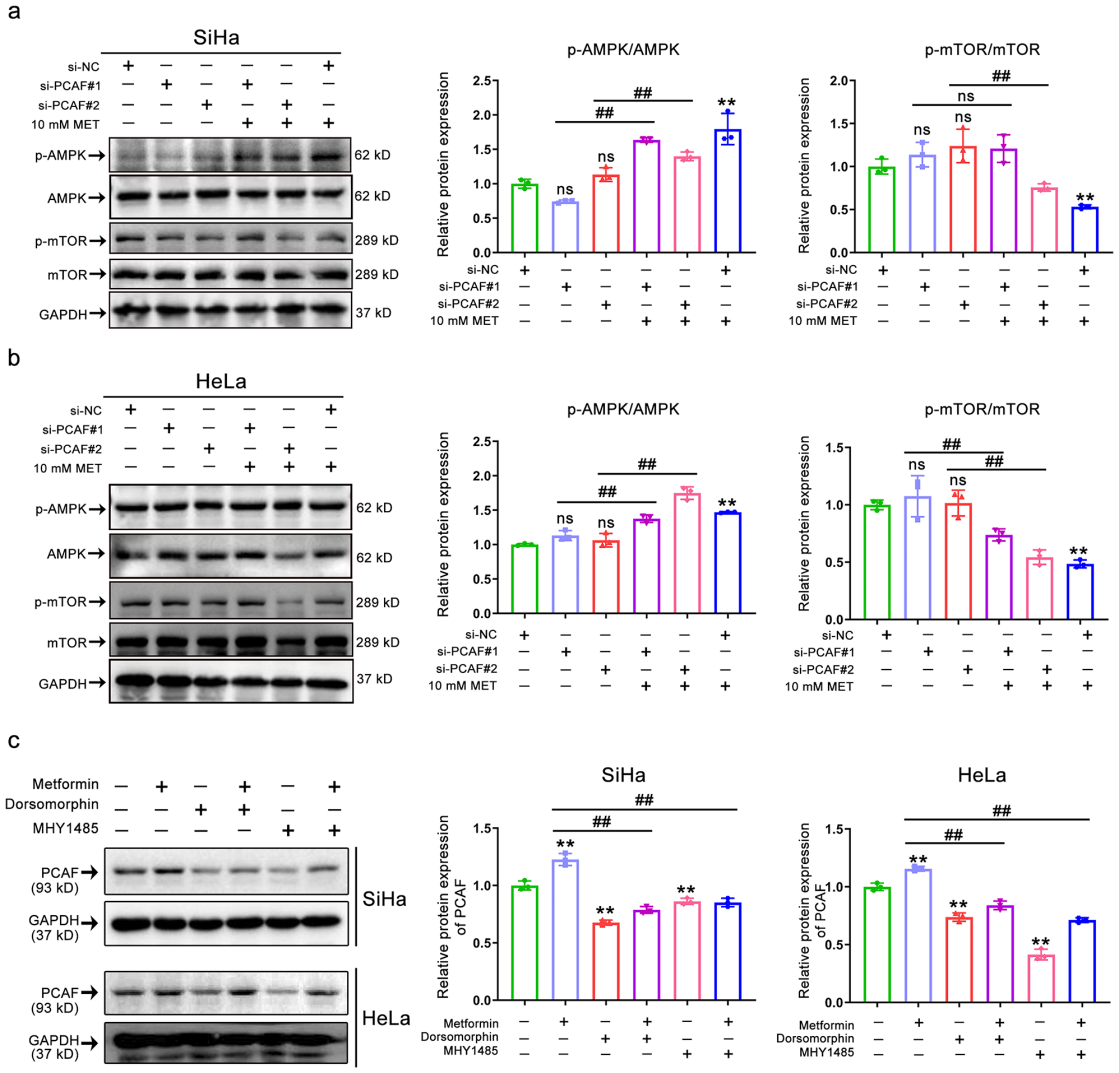
AMPK/mTOR axis participation in the regulation of PCAF acetylation activity. **(a-b)** Representative immunostaining images (left) and quantitative results (right) of the p-AMPK, AMPK, p-mTOR, and mTOR intensity in SiHa (a) and HeLa (b) cells after si-PCAF or (and) metformin (10 mM) treatment. **(c)** Representative immunostaining images (left) and quantitative results (right) of the PCAF intensity in SiHa (up) and HeLa (down) after dorsomorphin (10 µM) or (and) MHY1485 (10 µM) or (and) metformin (10 mM) treatment. Data represent means ± SD. *P* values were calculated by one-way ANOVA test. ns, not significant; ^#^*P* or ^*^P < 0.05; ^##^*P* or ^**^P < 0.01

The above results prompted us to further explore the molecular mechanism by which PCAF is regulated by the AMPK/mTOR axis. We hypothesized PCAF was a downstream protein of this axis. We then utilized siRNA for PCAF gene interference in HeLa and SiHa cells to determine whether PCAF expression affects AMPK and mTOR phosphorylation. The finding showed that the phosphorylation of AMPK and mTOR was not significantly altered after si-PCAF intervention, whereas it did change dramatically following metformin supplementation (Fig. 6a-b). To more precisely test this hypothesis, further in vitro experiments were conducted to explore whether blocking the AMPK/mTOR pathway affects the expression levels of PCAF. As shown in Fig. 6c, activation of AMPK by metformin upregulated PCAF expression, whereas blocking AMPK or activation of mTOR downregulated PCAF expression, leading us to speculate that PCAF is a downstream protein of the AMPK/mTOR axis. In addition, metformin-induced PCAF levels were abolished by the AMPK inhibitor dorsomorphin or the mTOR agonist MHY1485. Notably, in line with PCAF alterations, the trend in acetylation levels of H3K9ac also varied with changes in the AMPK/mTOR axis. Given that PCAF is an essential histone acetyltransferase for AMPK activation-induced H3K9 hyperacetylation, our findings suggest that the AMPK/mTOR axis regulates the acetylation activity of PCAF, which in turn modifies the acetylation level of H3K9.

### The validation of AMPK-PCAF-H3K9ac axis in vivo

**Fig. 7:**
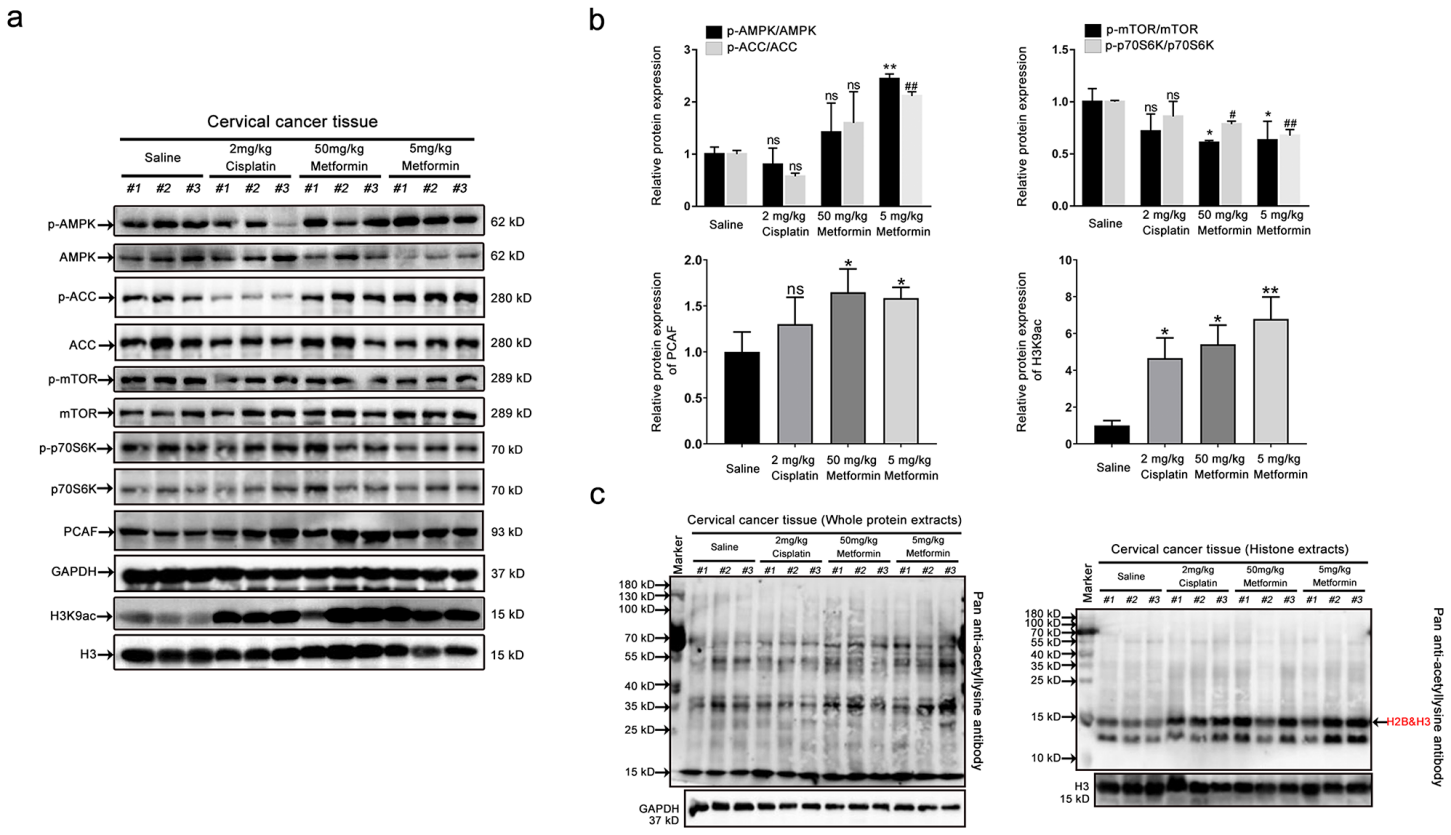
The validation of AMPK-PCAF-H3K9ac axis in vivo. (**a**) Representative Western blot images of the expression levels of p-AMPK, AMPK, p-ACC, ACC, p-mTOR, mTOR, p-p70S6K, p70S6K, PCAF, and H3K9ac in tumor tissues after cisplatin or metformin treatment. (**b**) Statistical analysis of the expression of p-AMPK/AMPK, p-ACC/ACC, p-mTOR/mTOR, p-p70S6K/p70S6K, PCAF, and H3K9ac. Data represent means ± SD (*n* = 3). *P* values were calculated by one-way ANOVA test. ns, not significant; ^#^*P* or ^*^P < 0.05; ^##^*P* or ^**^P < 0.01. (**c**) Representative Western blot images of acetylated protein expression levels in whole protein extracts (left) and whole histone extracts (right) of tumor tissues

To further explore whether the in vitro results of AMPK activation by metformin were consistent with the in vivo results, we examined the expression patterns of AMPK, ACC, mTOR, p70S6K, p-AMPK, p-ACC, p-mTOR, p-p70S6K, PCAF, and H3K9ac in tumor tissues collected from the cervical cancer xenograft nude mouse model. Metformin treatment significantly increased the levels of p-AMPK, p-ACC, PCAF, and H3K9ac in xenografts, while significantly decreasing the levels of p-mTOR and p-p70S6K (Fig. 7a-b). These findings are consistent with those at the cellular level, suggesting that in vivo activation of AMPK upregulates the acetylation capacity of PCAF to induce hyperacetylation of H3K9. Using a pan anti-acetylation immunoblot, we also evaluated the acetylation status of whole protein extracts and histone extracts from these tumor tissues. As depicted in Fig. 7c, activation of AMPK by metformin altered the acetylation levels of whole proteins, particularly whole histone H2B/H3. These results imply that the AMPK-PCAF-H3K9ac axis is crucial in the regulation of cervical cancer growth. Further and more in-depth research on this axis is warranted for future study.

### Genome-wide profile of H3K9ac targets in cervical cancer after activation of AMPK by metformin

**Fig. 8:**
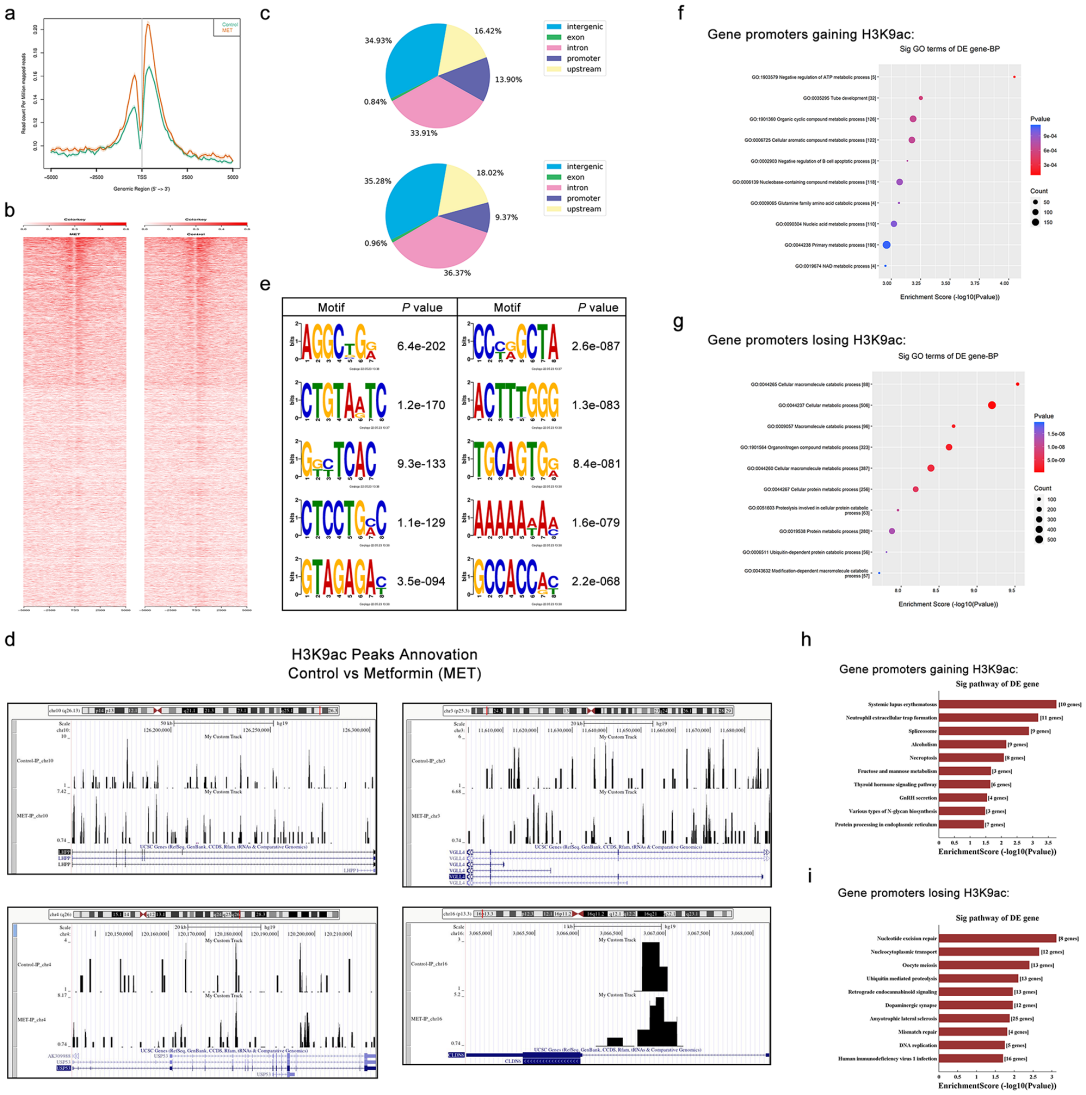
AMPK activation by metformin increase H3K9ac level at the transcription start site (TSS) of genes in SiHa. **(a)** ChIP-seq tag distribution of H3K9ac surrounding the TSS (± 5 kb) of the whole genome in the SiHa cells from control and metformin (MET) groups. **(b)** Heat maps of H3K9ac occupancy on the promoter region (TSS ± 5 kb), aligned by the degree of H3K9ac signal intensity in the control and metformin group. **(c)** Distribution of H3K9ac-binding regions across the genome in SiHa cells using ChIP-seq in the control and metformin groups. **(d)** Motif analysis of H3K9ac ChIP-seq data. **(e-f)** GO annotation of genes gaining H3K9ac **(e)** and genes losing this mark **(f)** of metformin treatment group vs. control. The dot plot shows the top 10 enrichment values of the significant enrichment terms involving biological process (BP). **(g-h)** KEGG pathway analysis of genes gaining H3K9ac **(g)** and losing this mark **(h)** in response to metformin treatment of SiHa cells. The bar plot shows the top 10 enrichment values of the significant enrichment terms involving KEGG pathways

ChIP-seq was performed on the SiHa cell line to identify the genes directly targeted by H3K9ac in response to AMPK activation by metformin. Consistent with the expression of H3K9ac protein, ChIP-seq results demonstrated that metformin treatment increased H3K9ac binding at gene transcription start sites (TSSs) relative to controls, indicating that AMPK activation increases chromatin accessibility for gene transcription (Fig. 8a-b). To elucidate AMPK-activation-induced differences in genome-wide H3K9ac binding, a comparison was conducted between the enriched peaks in the metformin group and the control group (Fig. 8c). The number of H3K9ac-enriched genes was significantly altered in metformin-treated cells compared to untreated cells. These peaks were extensively dispersed across genic (exon, intron, promoter, and upstream) and intergenic regions. Approximately 65% of H3K9ac peaks were found in genic regions, whereas the remaining H3K9ac peaks (~ 35%) were distributed throughout the intergenic regions. Differentially enriched region annotation identified that a total of 1,391 gene promoters (fold change > 2.0, *P* < 0.001) whose H3K9ac enrichments are significantly altered in cervical cancer treated with metformin, with 394 gaining H3K9ac, and 997 losing this mark in comparison to controls (Additional file 2). Among annotations of differentially enriched regions, we discovered that tumor suppressor genes such as LHPP, VGLL4, USP53, and CLDN6 had increased H3K9ac signals in their promoter regions under the metformin condition (Fig. 8d). These data suggest that activation of AMPK by metformin may promote the transcription of tumor suppressor genes by elevating H3K9ac levels, which in turn inhibits cervical cancer growth. Motif analysis of H3K9ac-binding regions showed differences in their binding sequence preferences (Fig. 8e).

Subsequently, we performed GO and KEGG pathway analysis for the putative target genes of H3K9ac. GO analysis revealed that these targets were involved in crucial metabolic and catabolic processes (Fig. 8f-g). According to the KEGG pathway analysis, gene promoters that gained H3K9ac were predominantly enriched in systemic lupus erythematosus, neutrophil extracellular trap formation, spliceosome, alcoholism, and necroptosis (Fig. 8h). Figure 8i reveals that gene promoters lacking H3K9ac were substantially enriched in nucleotide excision repair, nucleocytoplasmic transport, oocyte meiosis, ubiquitin mediated proteolysis, and retrograde endocannabinoid signaling.

## Discussion

Dysregulation of histone acetylation, an important epigenetic alteration closely associated with major pathologies including cancer, contributes to oncogenesis by inactivating tumor suppressor genes and activating oncogenic pathways [48, 49]. Global alterations in histone acetylation levels are relevant to a wide range of cancer phenotypes and have even been found to possess underlying prognostic value [26]. However, the molecular mechanisms that regulate histone acetylation remain largely unknown, particularly in the context of cervical cancer proliferation. Previous studies have found that metformin, a drug that is both low-priced and widely used, has a previously unrecognized role in enhancing histone acetylation, yet this effect has not been explored in cervical cancer [22, 23]. Metformin acts as an AMPK agonist to aid in cancer prevention and treatment [40]. Although the underlying molecular mechanism remains uncertain, it is hypothesized that metformin-induced histone modification through AMPK activation may be one such mechanism. Galdieri et al. [23] demonstrated that metformin hyperacetylated histones in ovarian cancer cells by activating AMPK. This suggests that AMPK is a crucial regulator of histone acetylation. The mechanisms by which AMPK controls histone acetylation and the specific histone lysine sites are targeted for acetylation, particularly in cervical cancer, remain unknown.

Herein we applied the AMPK agonist metformin and demonstrated that AMPK activation inhibits cervical cancer growth both in vitro and in vivo, accompanied by significant changes in the levels of acetylation modifications on both histones and non-histones. Given the complexity of global acetylation regulation, label-free quantitative acetylproteomics is required to determine the effects of AMPK activation on histone and non-histone acetylation, thereby identifying the precise lysine modification sites. To our knowledge, there have been no large-scale analyses of the effects of metformin-induced AMPK activation on the regulation of lysine acetylation during cervical carcinogenesis. Quantitative acetylproteomic results showed that 749 lysine acetylation sites on 509 proteins were differentially acetylated in the metformin-treated group in comparison to the control group. Following metformin treatment and subsequent AMPK activation, most non-histone acetylation levels were downregulated, whereas histone acetylation levels were upregulated, relative to controls. Among these, the acetylation sites on some proteins have been confirmed to play a role in the regulation of tumor progression. Our study showed that AMPK activation through metformin intervention resulted in the downregulation of acetylation at specific lysine residues (K139, K48, K275, K97, K323, and K220) of PGK1, an essential enzyme in the metabolic glycolytic pathway, compared to the control group. Hu et al. [50] discovered that PGK1 acts as an oncogenic factor and is involved in the progression of liver cancer, noting that acetylation of PGK1 at the K323 site is a key regulatory mechanism enhancing its enzymatic activity and metabolism in cancer cells. Additionally, NPM1, a multifunctional histone chaperone, is often overexpressed in various human cancers. Shandilya et al. [51] found that acetylation of NPM1 is positively correlated with tumorigenesis. Furthermore, the acetylation status of NPM1 could potentially be developed into a diagnostic marker and also serve as a potential therapeutic target for malignant transformation. Interestingly, our quantitative acetylproteomic results indicated that AMPK activation via metformin treatment significantly downregulated the acetylation levels at five lysine sites of NPM1 (K248, K202, K154, K229, and K141) in cervical cancer. In addition, the functions of the acetylation sites on some proteins discovered in this study have not been reported in the literature. However, the acetylation of these proteins has been confirmed to play a role in the regulation of tumor development, including SDHA [52], IDH1 [53], and HMGB1 [54]. It can be seen that the regulation of AMPK activation in cervical cancer progression involves a large and complex regulatory network of protein acetylation, much of which remains to be elucidated.

Disruption of the histone acetylation balance leads to unregulated activation of numerous genes, resulting in events associated with malignant transformation. Numerous investigations have linked changes in histone acetylation as promising diagnostic or prognostic indicators in cancer. Notably, our quantitative acetylproteomic data and Western blot experiments have shown that AMPK activation by metformin dramatically increases acetylation at six lysine sites in histones H2B (K20, K23, K12) and H3 (K122, K56, K9) in cervical cancer cells. However, the function of these histone acetylation modifications in cancer progression remain not fully elucidated. In particular, H3K56ac, H3K122ac, H2BK12ac, H2BK20ac, and H2BK23ac—rarely studied histone modifications—have many gaps to fill regarding their roles in tumors. For H3K9ac, although more studies have been reported, further investigation is still needed. For example, current studies suggest that the role of H3 acetylation at K9 in tumors is a matter of debate. Liana et al. [29] demonstrated that hypoacetylation of H3K9ac is a poor prognosis marker in oral cancer; while hyperacetylation of H3K9ac acts as a transcriptional activator, enhancing various signaling pathways to promote tumorigenesis [55]. Additionally, elevated H3K9ac has been associated with a poor prognosis in cervical cancer [56], while in glioma, it is associated with a better prognosis [57]. Consequently, the aim of this study is not only to elucidate the role of H3K9ac in cervical cancer growth but also to investigate the molecular mechanisms that modulate H3K9 acetylation.

There are numerous downstream target genes regulated by H3K9ac, and ChIP-seq was utilized to identify the potential downstream molecules. With the aid of this technology, we were able to identify alterations in H3K9ac binding to DNA resulting from metformin-induced AMPK activation. Based on a comprehensive analysis of ChIP-seq data, we targeted genes with elevated H3K9ac levels in the TSS region. Their representative genes include the tumor suppressor genes LHPP, VGLL4, USP53, and CLDN6, which have been reported to play significant anti-tumor functions in a variety of cancer types. Overexpression of LHPP inhibited the proliferation, migration, and invasion of cervical cancer cells, which was accompanied by modifications to the p53 and metastatic signaling pathways [58]. VGLL4 suppresses the cancerous phenotype of malignant epidermal squamous cell carcinoma by inhibiting YAP1/TEAD-dependent pro-cancer signaling [59]. Cheng et al. [60] reported that USP53 inhibited the proliferation and growth of esophageal carcinoma cells in vitro and in vivo, and that USP53 activation by H3K27 acetylation modulates cell viability via the AMPK signaling pathway. Lu et al. [61] identified that overexpression of CLDN6 inhibited the proliferation of breast cancer cells and demonstrated that this effect was mediated by the inhibition of ERK/Sp1/cyclin D1 and ERK/IL-8 signaling. We hypothesize that AMPK activation induces H3K9 acetylation, leading to chromatin remodeling and transcriptional activation in cervical cancer; H3K9ac binds to the promoter regions of certain tumor suppressor DNAs and enhances their expression, thereby inhibiting the growth of cervical cancer. Therefore, targeting these genes may offer new perspectives for H3K9ac-related activation of AMPK anti-cervical cancer research.

In our study, we discovered that AMPK activation induces H3K9 hyperacetylation and inhibits cervical cancer growth in vivo and in vitro; this mechanism may be facilitated by an AMPK-dependent mechanism mediated by mTOR signaling inhibition. Dorsomorphin (AMPK inhibitor) and MHY1485 (mTOR agonist) are frequently used as small-molecule tools to study the role of AMPK/mTOR signaling [62], and dorsomorphin, in particular, has been shown to block AMPK/mTOR signaling [63]. In addition, studies have shown that major molecular targets of metformin included AMPK and mTOR [64]. We therefore hypothesize that H3K9ac is regulated by the AMPK/mTOR axis. However, the regulation of this process requires additional research.

**Fig. 9:**
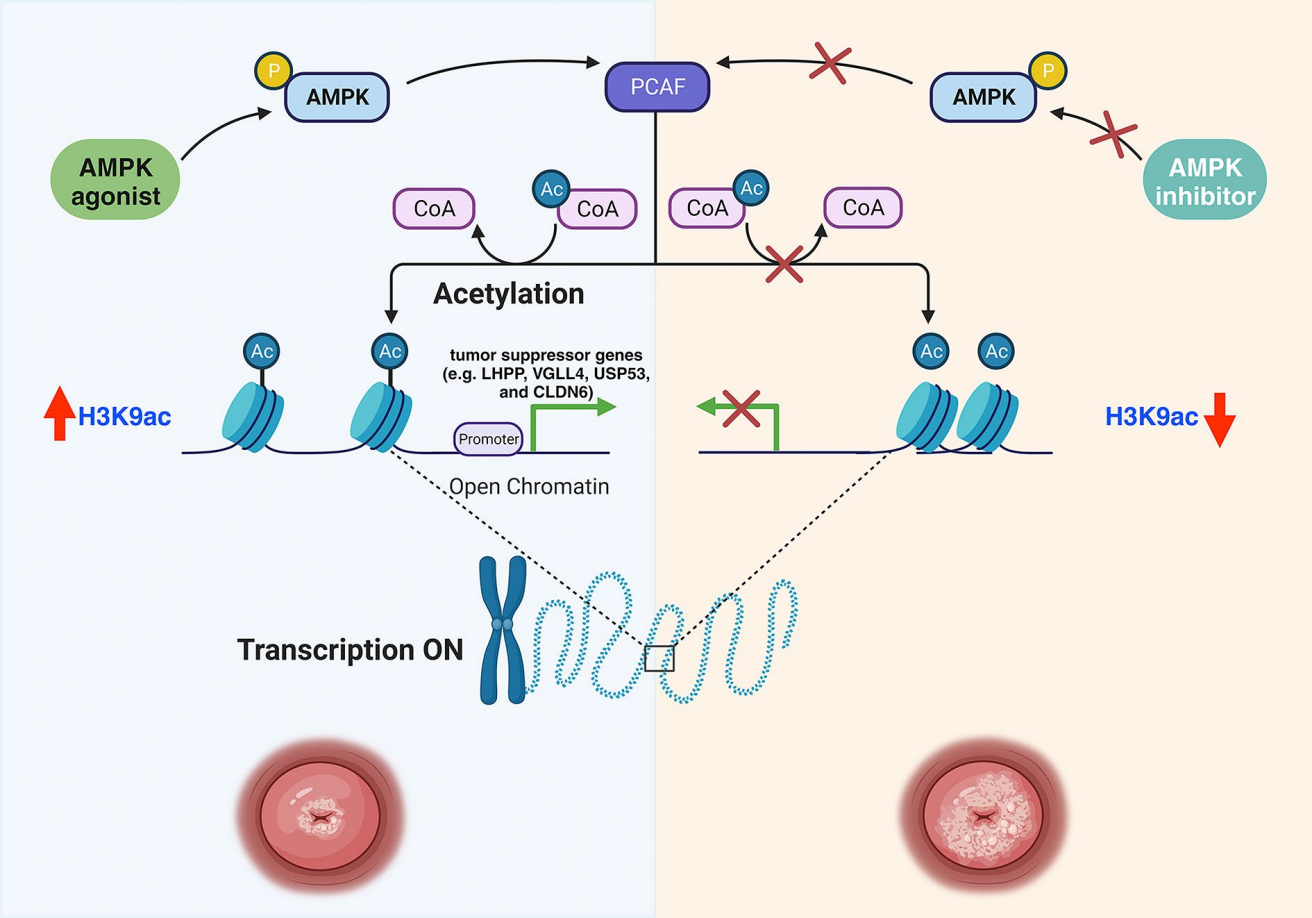
Schematic diagram of the hypothesized molecular mechanism of AMPK activation by metformin against cervical cancer growth in this study

Histone acetylation is a dynamic and reversible procedure that is mediated by lysine deacetylases and lysine acetyltransferases. PCAF, belonging to the GNAT family of lysine acetyltransferase, acetylates histone H3K9 [65]. We demonstrated that activation or inhibition of AMPK increases or decreases PCAF expression, respectively, which in turn modifies H3K9ac levels. This suggests that PCAF is involved in the acetylation of H3K9 in cervical cancer. In PCAF-deficient cervical cancer cell lines, supplementation with metformin was ineffective at restoring H3K9ac levels, indicating that AMPK activation loses its capacity to hyperacetylate H3K9 in the absence of PCAF. Consequently, PCAF is indispensable for AMPK activation-induced H3K9 hyperacetylation. Moreover, using the GEO database, we described the expression pattern of PCAF in cervical cancer and normal cervical epithelial tissues. The results were consistent with the findings of Li et al. [47], who discovered that the expression of PCAF in cervical cancer tissues was lower than that in the control group. In addition, our in vitro proliferation assay revealed that changes in PCAF expression impact the survival of cervical cancer cell lines; low PCAF expression may promote tumor cell survival. We hypothesize that PCAF-mediated H3K9 acetylation is an important mechanism for regulating the proliferation of cervical cancer. Furthermore, we hypothesize that PCAF is required for AMPK-mediated H3K9 hyperacetylation and that the AMPK-PCAF-H3K9ac axis plays an essential role in regulating the proliferation of cervical cancer. As depicted in Fig. 9, we postulate a molecular mechanism whereby AMPK-activated PCAF can inhibit cervical cancer growth by hyperacetylating H3K9.

## Conclusions

Using the AMPK agonist metformin, this study demonstrated that AMPK activation can epigenetically induce H3K9 acetylation and that the acetyltransferase PCAF is necessary for the hyperacetylation of H3K9 induced by AMPK activation. In addition, H3K9 acetylation leads to chromatin remodeling and transcriptional activation in cervical cancer, enhancing the binding to promoter regions of specific tumor-suppressor genes and thus activating their expression, which in turn inhibits the growth of cervical cancer. Thus, our study not only elucidates the role of the AMPK-PCAF-H3K9ac axis in the development of cervical cancer but also supports the clinical application of metformin inhibiting tumorigenesis and cancer progression.

### Electronic supplementary material

Below is the link to the electronic supplementary material.

Supplementary Material 1

Supplementary Material 2

Supplementary Material 3

Supplementary Material 4

Supplementary Material 5

Supplementary Material 6

## Data Availability

The authors declare that all the data supporting the findings of this study are available within the article and its Supplemental information files. Publicly available datasets were analyzed in this study. This data can be found in GEO.

## Abbreviations

AMPK: AMP-activated protein kinase
TMT: Tandem mass tag
hrHPV: High-risk human papillomavirus
HAT: Histone acetyltransferase
HDAC: Histone deacetylase
DMEM: Dulbecco’s modified Eagle’s medium
PS: Penicillin-streptomycin
DMSO: Dimethyl sulfoxide
CST: Cell Signaling Technology
MET: Metformin
CIS: Cisplatin
CCK-8: Cell counting kit-8
SPF: Specific pathogen-free
PMSF: Phenylmethylsulfonyl fluoride
SDS-PAGE: Sodium dodecyl sulfate–Polyacrylamide Gel Electrophoresis
PVDF: Polyvinylidene Fluoride
DTT: Dithiothreitol
IAA: IODOACETAMIDE
TEAB: Triethylammonium Bicarbonate
NETN: Buffer comprising 100 mM NaCl, 1 mM EDTA, 50 mM Tris-HCl, and 0.5% NP-40
TFA: Trifluoroacetic Acid
LC-MS/MS: Liquid Chromatography-Tandem Mass Spectrometry
UPLC: Ultra-Performance Liquid Chromatography
NSI: Nanospray Ionization
GO: Gene Ontology
KEGG: Kyoto Encyclopedia of Genes and Genomes
GEO: Gene Expression Omnibus
FDR: False Discovery Rate
SD: Standard Deviation
Kac: Lysine Acetylation
PCAF: P300/CBP-associated factor
PGK1: Phosphoglycerate Kinase 1
NPM1: Nucleophosmin
GNAT: GCN5-related N-acetyltransferase
ChIP-seq: Chromatin Immunoprecipitation-Sequencing
