# Correction: Activation of AMPK inhibits cervical cancer growth by hyperacetylation of H3K9 through PCAF

**DOI:** 10.1186/s12964-024-01702-x

**Published:** 2024-06-13

**Authors:** Botao Pan, Can Liu, Jiyan Su, Chenglai Xia

**Affiliations:** 1 Foshan Women and Children Hospital, Foshan, 528000 China; 2 School of Pharmaceutical Sciences, Southern Medical University, Guangzhou, 515150 China

**Correction: Cell Commun Signal 22, 306 (2024)**

10.1186/s12964-024-01687-7

Following the publication of original article [1], the authors noticed an error in the order of the figures in their published article. The correct order of figures is as follows:

**Fig. 1:**
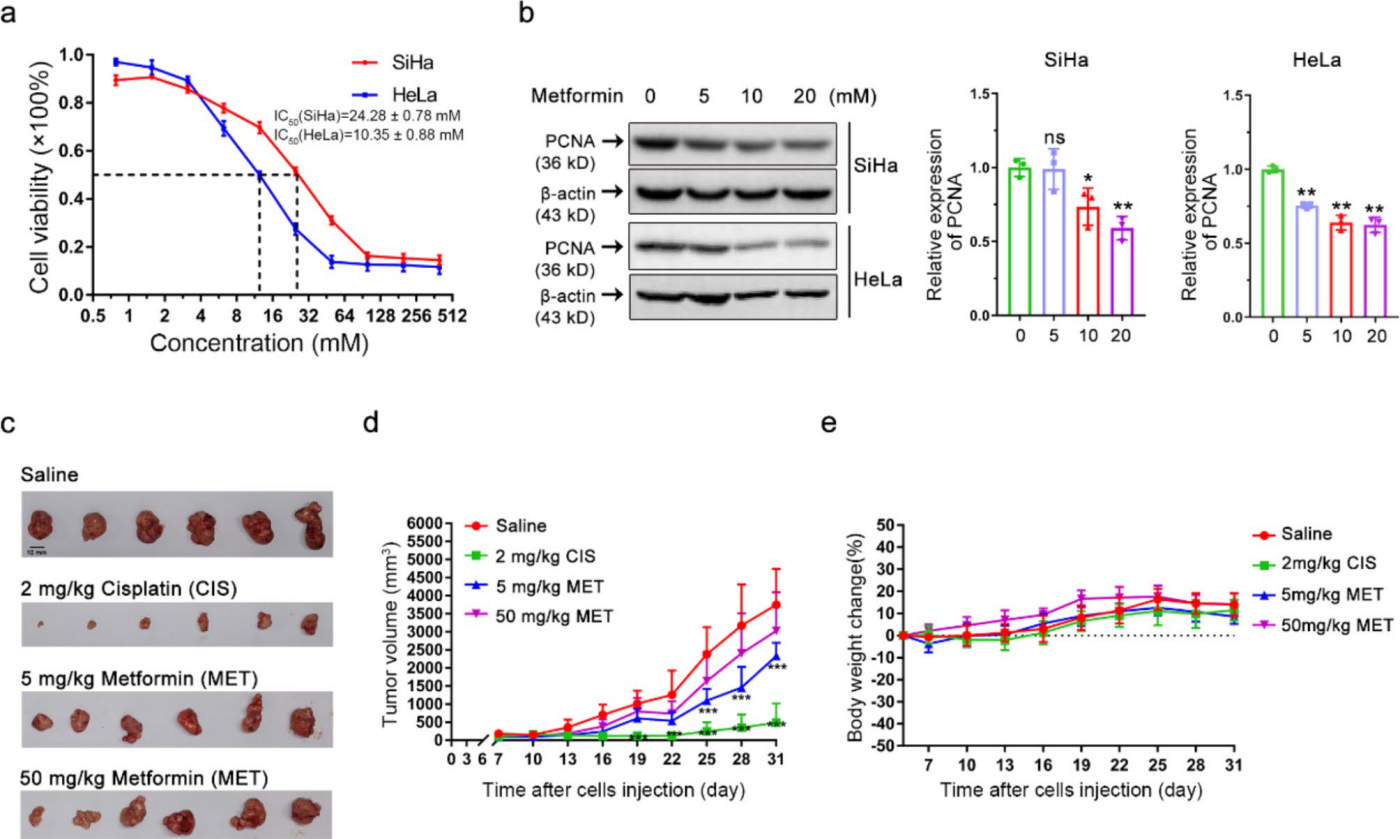
Metformin, an AMPK agonist, exerts cytotoxic and anti-tumor effects on cervical cancer. **(a)** The cell viability of HeLa and SiHa cells following metformin treatment was determined by CCK-8 assay. Data are means ± SD (*n* = 3). **(b)** Western blot analysis was performed to assess PCNA protein levels in cervical cancer cell lines HeLa and SiHa following metformin treatment (left). A statistical analysis of the expression of PCNA (right). Data represent means ± SD (*n* = 3). *P* values were calculated by one-way ANOVA. ^*^P < 0.05, ^**^P < 0.01, ns, not significant. **(c)** SiHa cell xenograft tumors from various groups, saline control group, 2 mg/kg cisplatin (CIS), 5 mg/kg metformin (MET), and 50 mg/kg metformin (MET). **(d)** Xenograft tumor growth curves of SiHa cells from different treatment groups. Tumor volumes formed in BALB/c nude mice were measured every 3 days after implantation of SiHa cells. Data represent means ± SD (*n* = 6). *P* values were calculated by multiple *t*-test. ^*^P < 0.05, ^**^P < 0.01, ^***^P < 0.001. **(e)** Body weight change (%) curves of mice in different treatment groups. The curves were plotted by collecting mouse weight data every 3 days after the implantation of SiHa cells (*n* = 6)

**Fig. 2:**
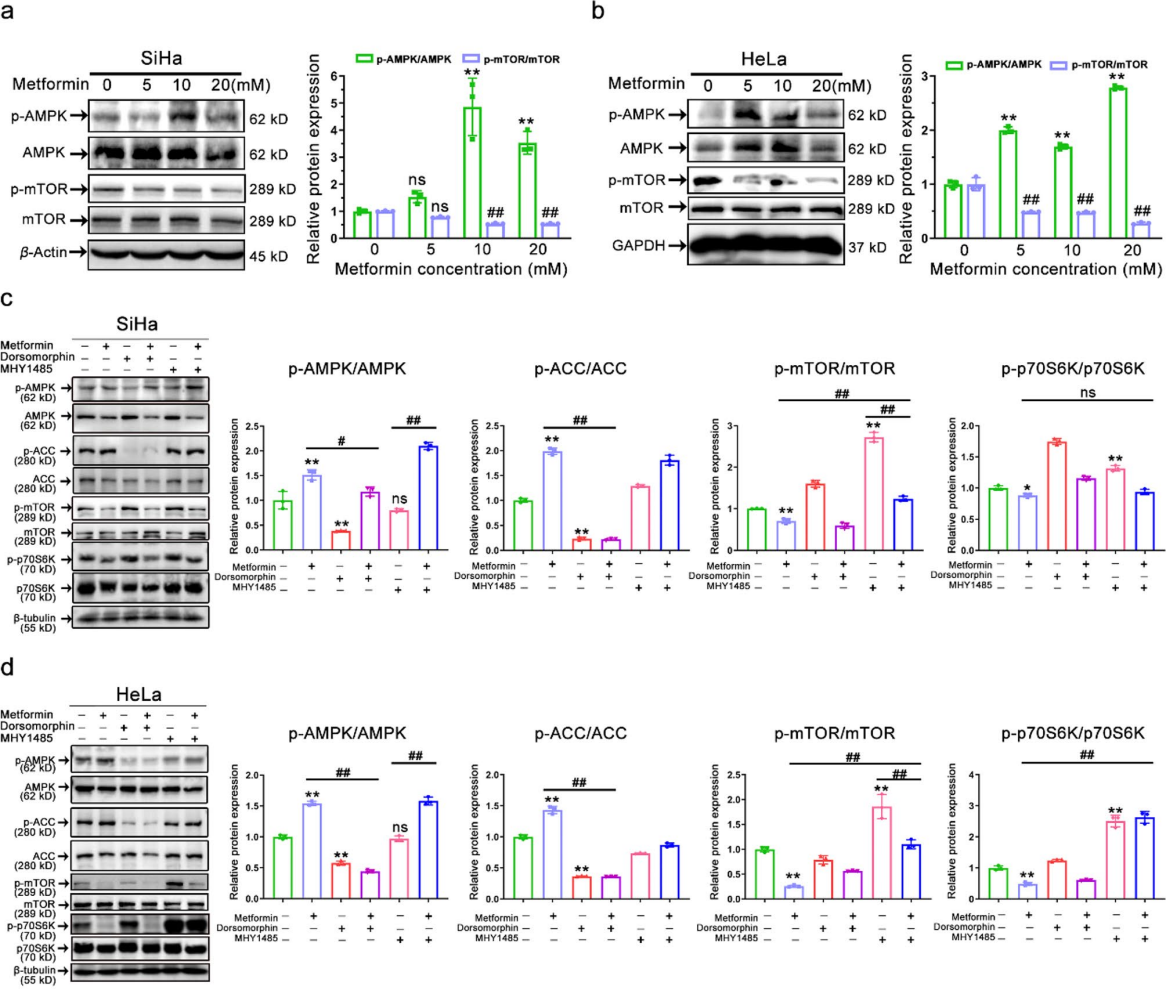
Effect of AMPK agonist metformin on the AMPK/mTOR pathway. (**a-b**) Representative immunostaining images (left) and their corresponding quantitative results (right) of the intensities of p-AMPK, AMPK, p-mTOR, and mTOR in metformin-treated (0 ∼ 20 mM) SiHa (a) and HeLa (b) cells for 48 h. Data represent means ± SD (*n* = 3). *P* values were calculated using a one-way ANOVA test. ns, not significant; ^#^*P* or ^*^P < 0.05, ^##^*P* or ^**^P < 0.01 (vs. control group). **(c-d)** Representative immunostaining images (left) and their corresponding quantitative results (right) of the intensities of p-AMPK, AMPK, p-ACC, ACC, p-mTOR, mTOR, p-p70S6K, and p70S6K in SiHa (c) and HeLa (d) cells following treatment with dorsomorphin (10 µM), MHY1485 (10 µM), or metformin (10 mM). Data represent means ± SD (*n* = 3). *P* values were calculated by one-way ANOVA test. ns, not significant; ^#^*P* or ^*^P < 0.05, ^##^*P* or ^**^P < 0.01

**Fig. 3:**
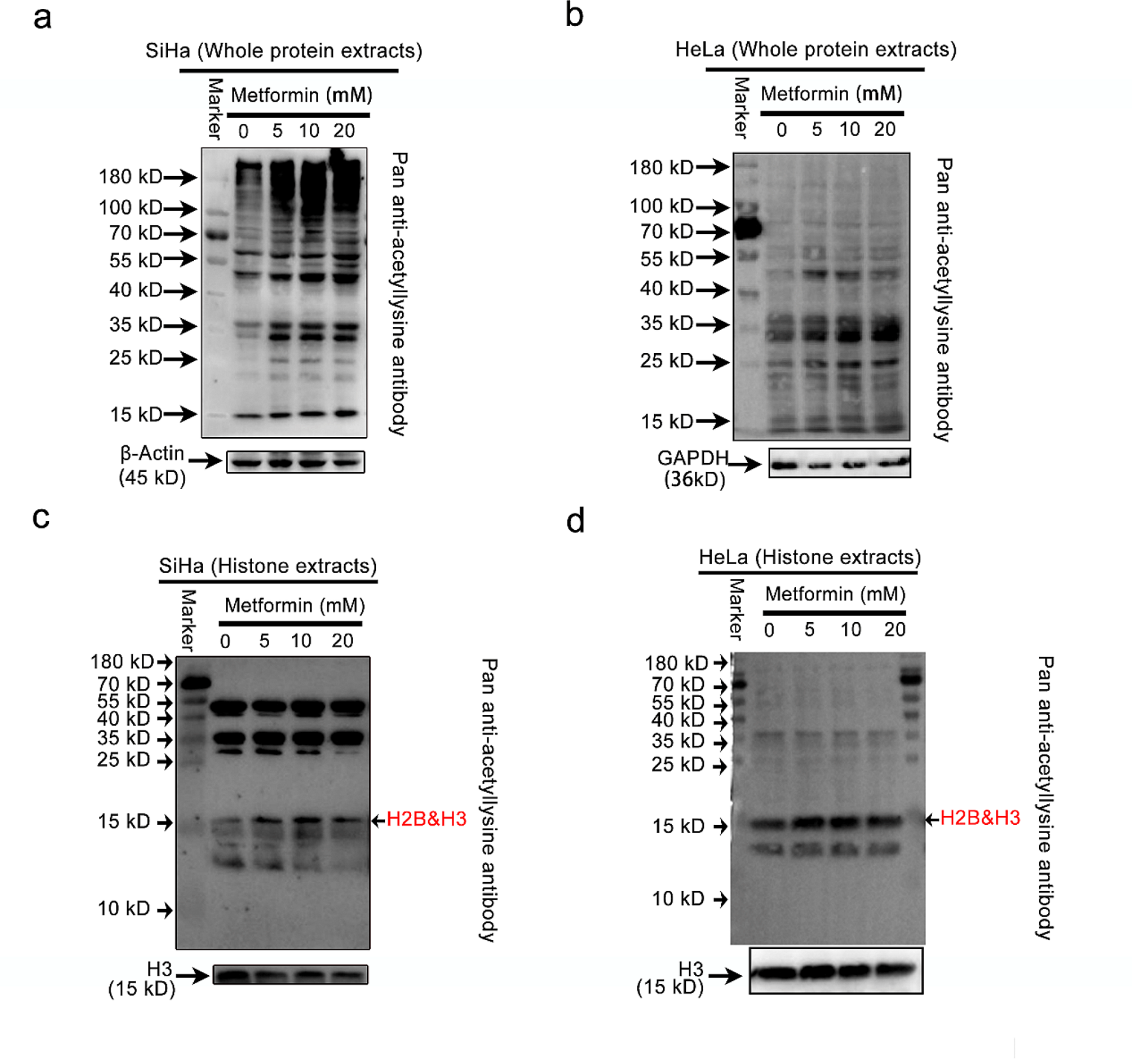
Profiling the global lysine acetylation of proteins through AMPK activation. **(a-b)** Representative Western blot images of the expression levels of acetylated proteins in whole protein extracts of SiHa **(a)** and HeLa **(b)** after metformin treatment. **(c-d)** Representative Western blot images of the expression levels of acetylated proteins in whole histone extracts of SiHa **(c)** and HeLa **(d)** after metformin treatment

**Fig. 4:**
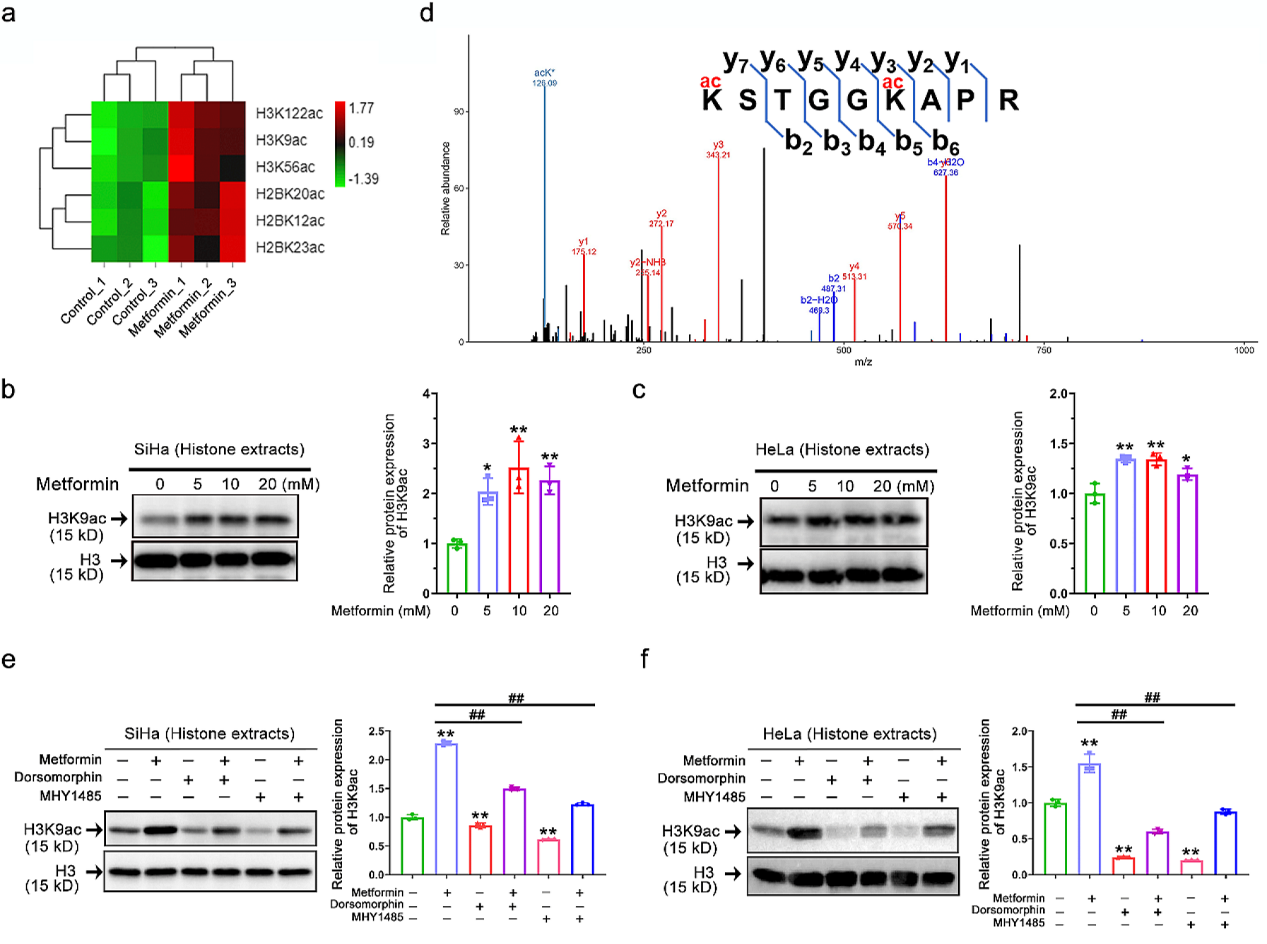
AMPK activation induces H3K9 hyperacetylation in vitro. **(a)** Heatmap analysis of metformin-altered acetylation levels at different lysine sites on histone H3 and H2B in cervical cancer cells based on quantitative acetylproteomic results (*n* = 3). **(b-c)** Representative immunostaining images (left) and quantitative results (right) of the H3K9ac intensity in whole histone extracts of SiHa **(b)** and HeLa **(c)** after metformin (0 ∼ 20 mM) treatment. Data represent means ± SD (*n* = 3). *P* values were calculated by one-way ANOVA. ^*^P < 0.05, and ^**^P < 0.01. **(d)** Identification by LC-MS/MS of H3 4–12 peptides carrying acetylation (ac) at K9. **(e-f)** Representative immunostaining images (left) and quantitative results (right) of the H3K9ac intensity in SiHa (e) and HeLa (f) after dorsomorphin (10 µM) or (and) MHY1485 (10 µM) or (and) metformin (10 mM) treatment. Data represent means ± SD (*n* = 3). *P* values were calculated by one-way ANOVA. ^#^*P* or ^*^P < 0.05, ^##^*P* or ^**^P < 0.01

**Fig. 5:**
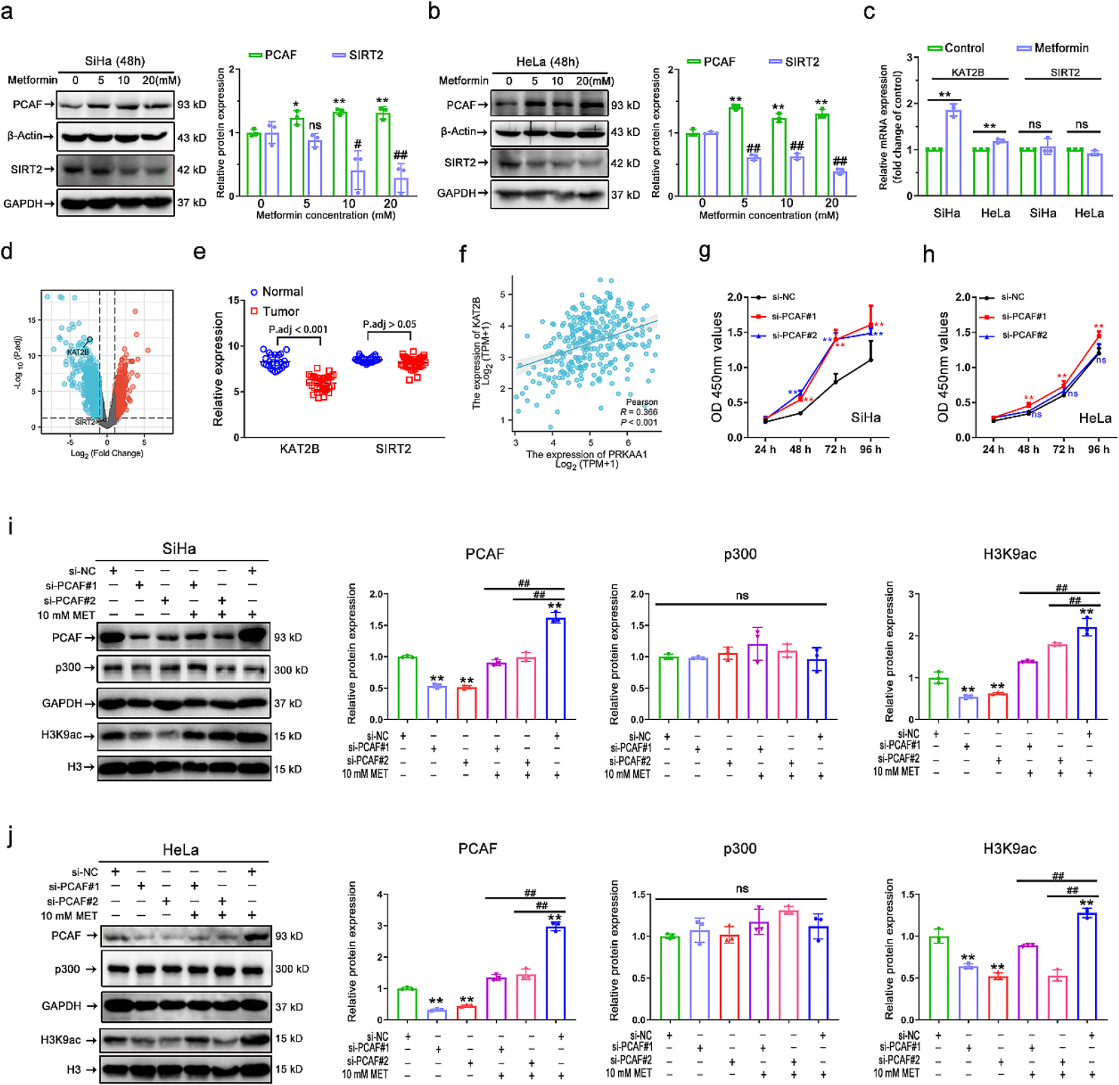
PCAF is essential for AMPK-activation-induced H3K9 hyperacetylation. **(a-b)** Representative immunostaining images (left) and quantitative results (right) of the PCAF and SIRT2 intensity in metformin (0 ∼ 20 mM) treated SiHa (a) and HeLa (b) cells. **(c)** Relative mRNA expression levels of KAT2B, and SIRT2 in SiHa and HeLa after 10 mM metformin treatment (*n* = 3). **(d)** Volcano plots of significantly differentially expressed genes identified in the GEO database between 33 primary cervical cancer tissues and 24 normal cervical epithelial tissues. **(e)** Dot plots of the relative expression of KAT2B and SIRT2 genes between 33 primary cervical cancer tissues and 24 normal cervical epithelial tissues. **(f)** Correlation Analysis of AMPK (encoded by PRKAA1) and PCAF (encoded by KAT2B) Expression in the TCGA-CESC Dataset. **(g-h)** Cell growth curves under different treatments of si-NC, si-PCAF#1, and si-PCAF#2 in SiHa **(g)** and HeLa **(h)** cells (*n* = 3). (**i-j**) Representative immunostaining images (left) and quantitative results (right) of the PCAF, p300, and H3K9ac intensity in SiHa (i) and HeLa (j) cells after si-PCAF or (and) metformin (10 mM) treatment. Data are means ± SD. The two-tailed Student’s *t*-test (c) and one-way ANOVA (a/b/i/j) were used to performed comparison between two groups and more groups, respectively. For g/h, *P* values were calculated by multiple *t*-test. ns, not significant; ^#^*P* or ^*^P < 0.05; ^##^*P* or ^**^P < 0.01

**Fig. 6:**
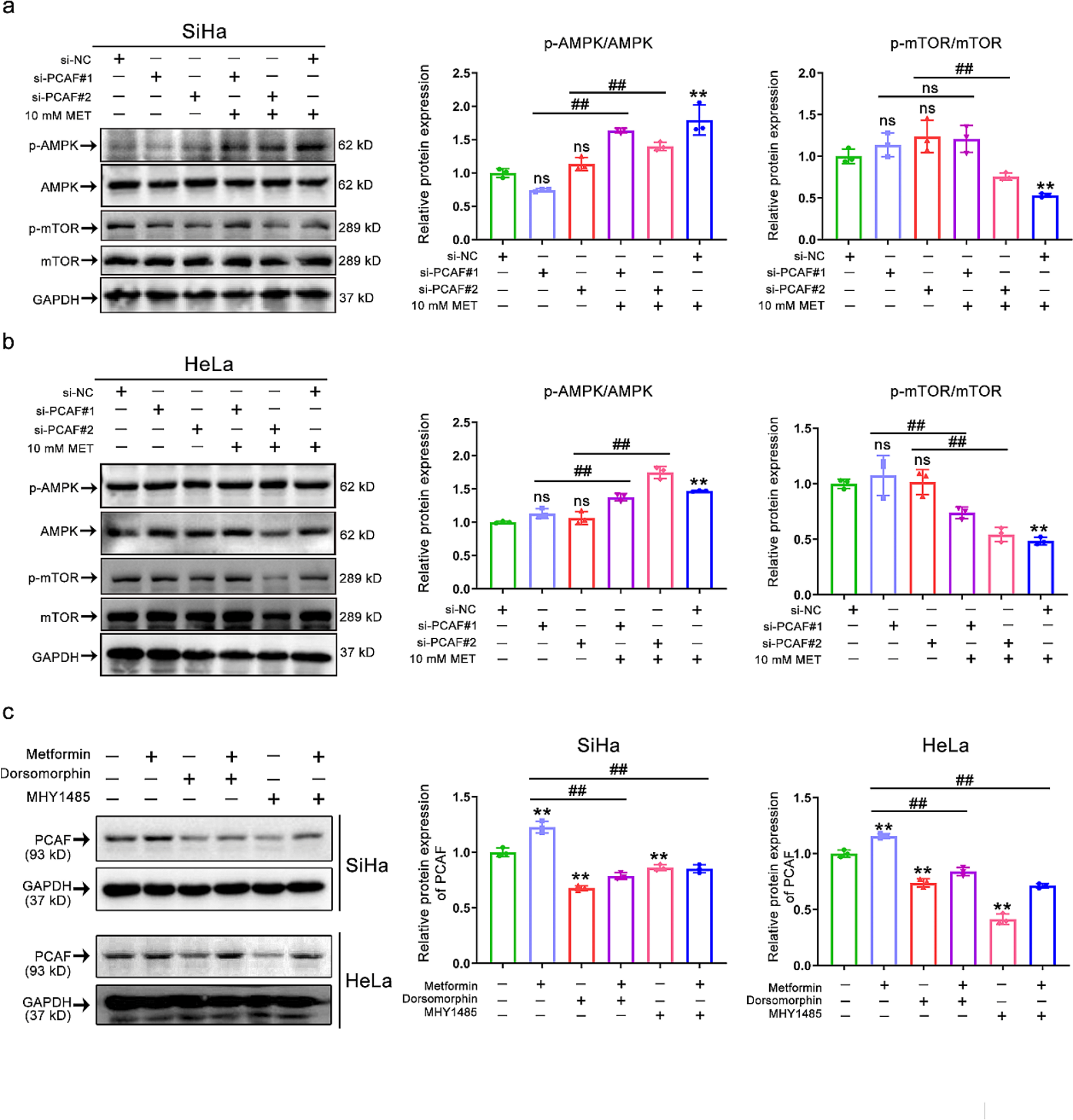
AMPK/mTOR axis participation in the regulation of PCAF acetylation activity. **(a-b)** Representative immunostaining images (left) and quantitative results (right) of the p-AMPK, AMPK, p-mTOR, and mTOR intensity in SiHa **(a)** and HeLa **(b)** cells after si-PCAF or (and) metformin (10 mM) treatment. **(c)** Representative immunostaining images (left) and quantitative results (right) of the PCAF intensity in SiHa (up) and HeLa (down) after dorsomorphin (10 µM) or (and) MHY1485 (10 µM) or (and) metformin (10 mM) treatment. Data represent means ± SD. *P* values were calculated by one-way ANOVA test. ns, not significant; ^#^*P* or ^*^P < 0.05; ^##^*P* or ^**^P < 0.01

**Fig. 7:**
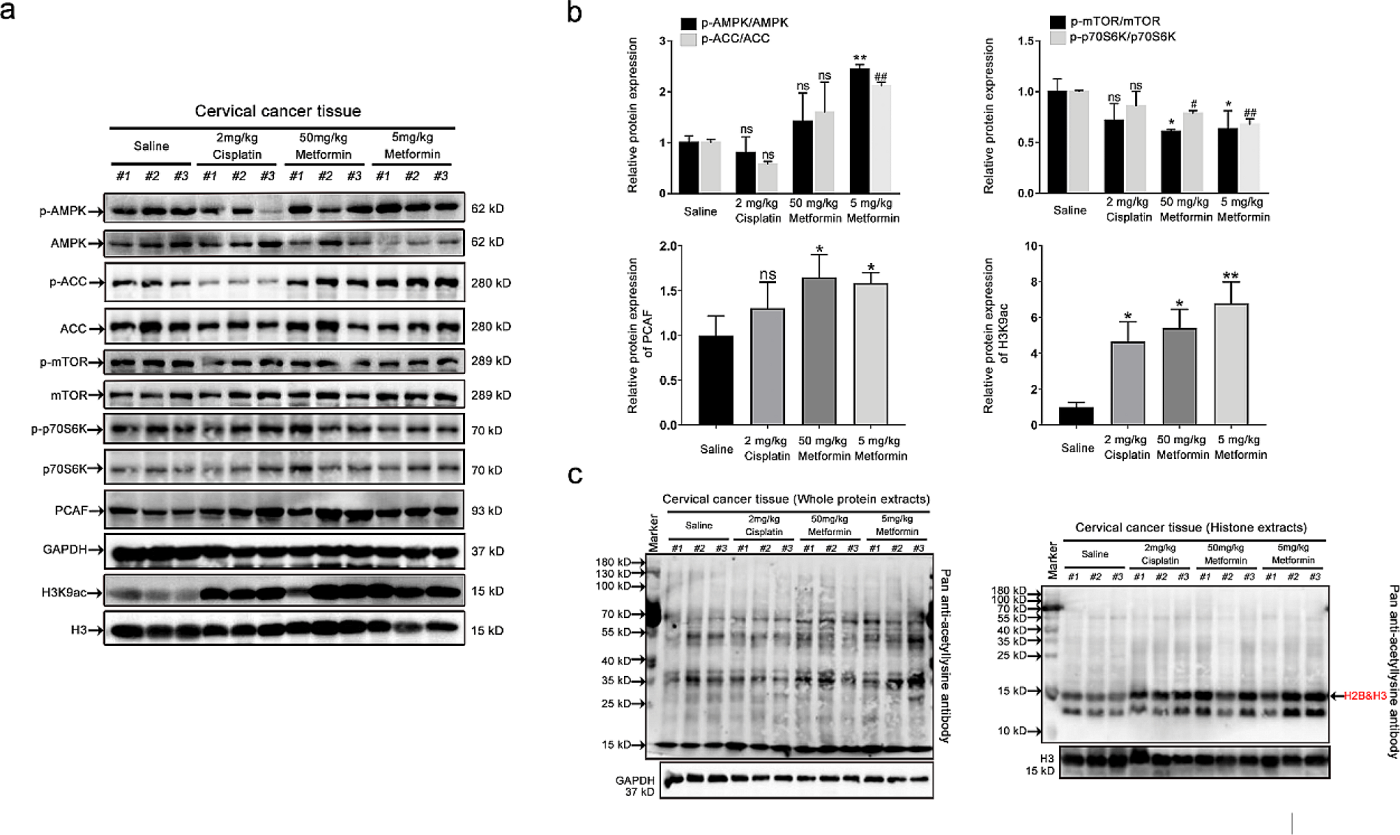
The validation of AMPK-PCAF-H3K9ac axis in vivo. (**a**) Representative Western blot images of the expression levels of p-AMPK, AMPK, p-ACC, ACC, p-mTOR, mTOR, p-p70S6K, p70S6K, PCAF, and H3K9ac in tumor tissues after cisplatin or metformin treatment. (**b**) Statistical analysis of the expression of p-AMPK/AMPK, p-ACC/ACC, p-mTOR/mTOR, p-p70S6K/p70S6K, PCAF, and H3K9ac. Data represent means ± SD (*n* = 3). *P* values were calculated by one-way ANOVA test. ns, not significant; ^#^*P* or ^*^P < 0.05; ^##^*P* or ^**^P < 0.01. (**c**) Representative Western blot images of acetylated protein expression levels in whole protein extracts (left) and whole histone extracts (right) of tumor tissues

**Fig. 8:**
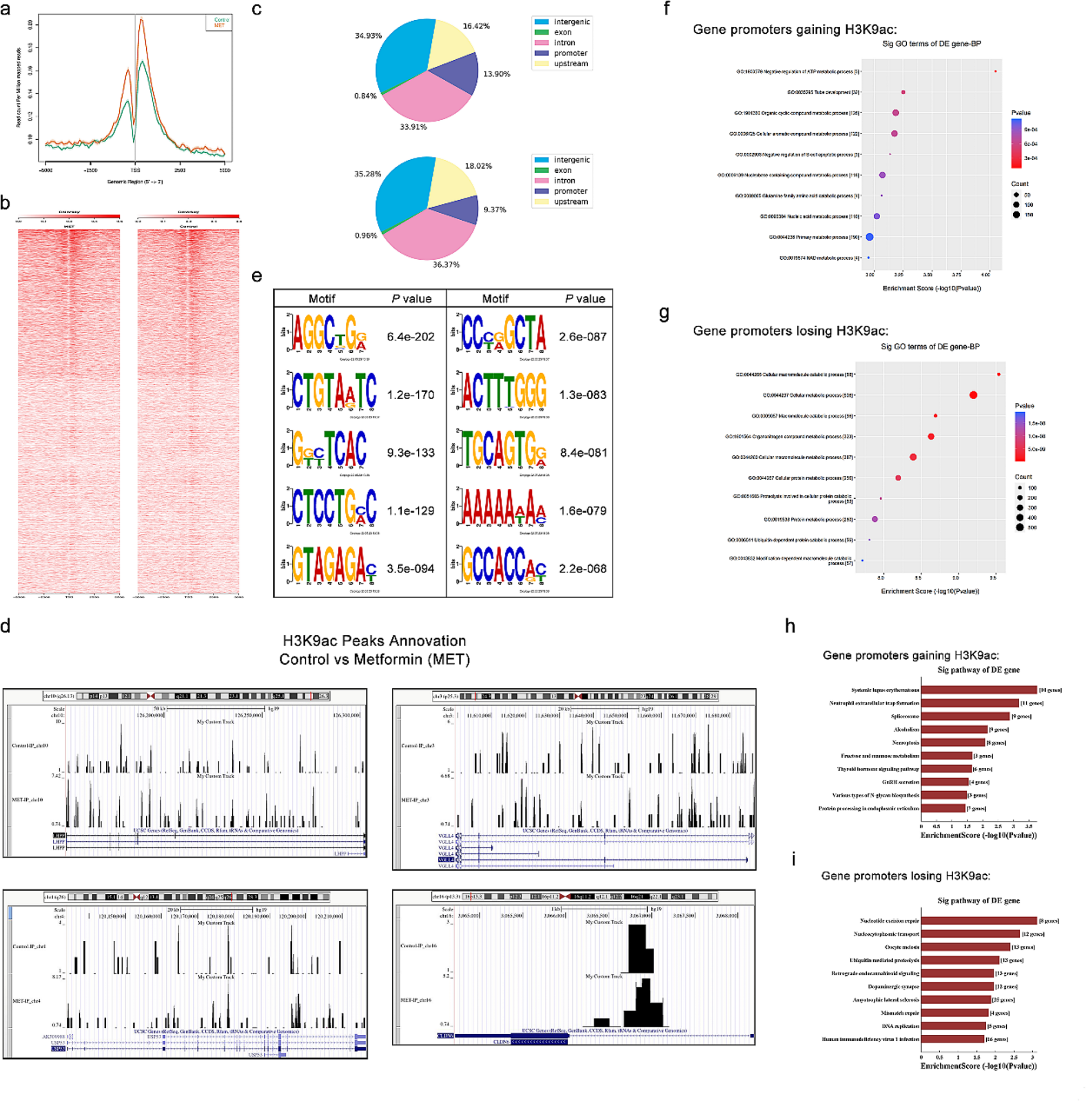
AMPK activation by metformin increase H3K9ac level at the transcription start site (TSS) of genes in SiHa. **(a)** ChIP-seq tag distribution of H3K9ac surrounding the TSS (± 5 kb) of the whole genome in the SiHa cells from control and metformin (MET) groups. **(b)** Heat maps of H3K9ac occupancy on the promoter region (TSS ± 5 kb), aligned by the degree of H3K9ac signal intensity in the control and metformin group. **(c)** Distribution of H3K9ac-binding regions across the genome in SiHa cells using ChIP-seq in the control and metformin groups. **(d)** Motif analysis of H3K9ac ChIP-seq data. **(e-f)** GO annotation of genes gaining H3K9ac **(e)** and genes losing this mark **(f)** of metformin treatment group vs. control. The dot plot shows the top 10 enrichment values of the significant enrichment terms involving biological process (BP). **(g-h)** KEGG pathway analysis of genes gaining H3K9ac **(g)** and losing this mark **(h)** in response to metformin treatment of SiHa cells. The bar plot shows the top 10 enrichment values of the significant enrichment terms involving KEGG pathways

**Fig. 9:**
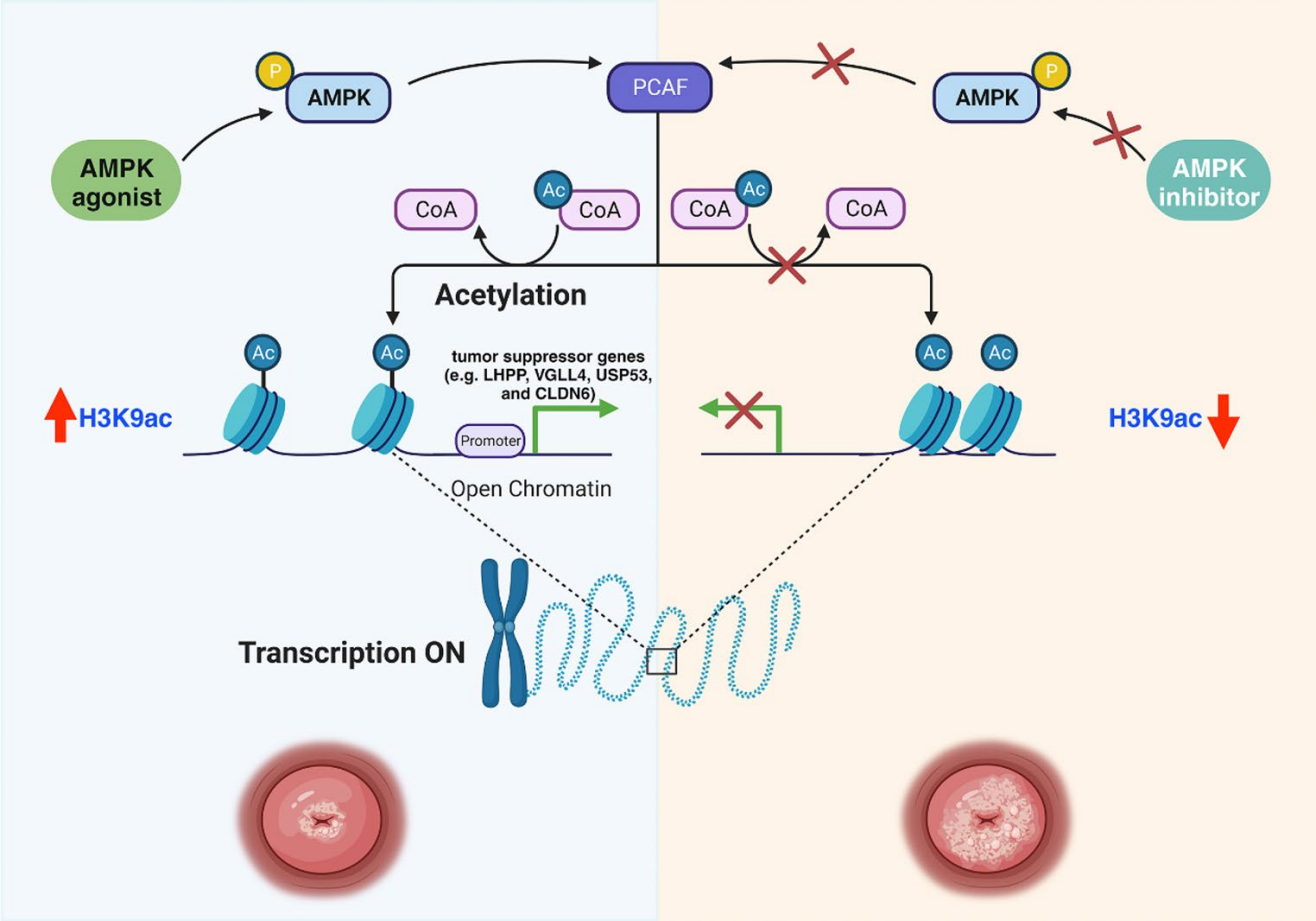
Schematic diagram of the hypothesized molecular mechanism of AMPK activation by metformin against cervical cancer growth in this study

The original article [1] has been updated.
