# Supplementary figures and images for: Activation of AMPK inhibits cervical cancer growth by hyperacetylation of H3K9 through PCAF
Source: Cell Commun Signal. 2024 Jun 3;22:306. doi: 10.1186/s12964-024-01687-7 (PMC11145780; doi:10.1186/s12964-024-01687-7)

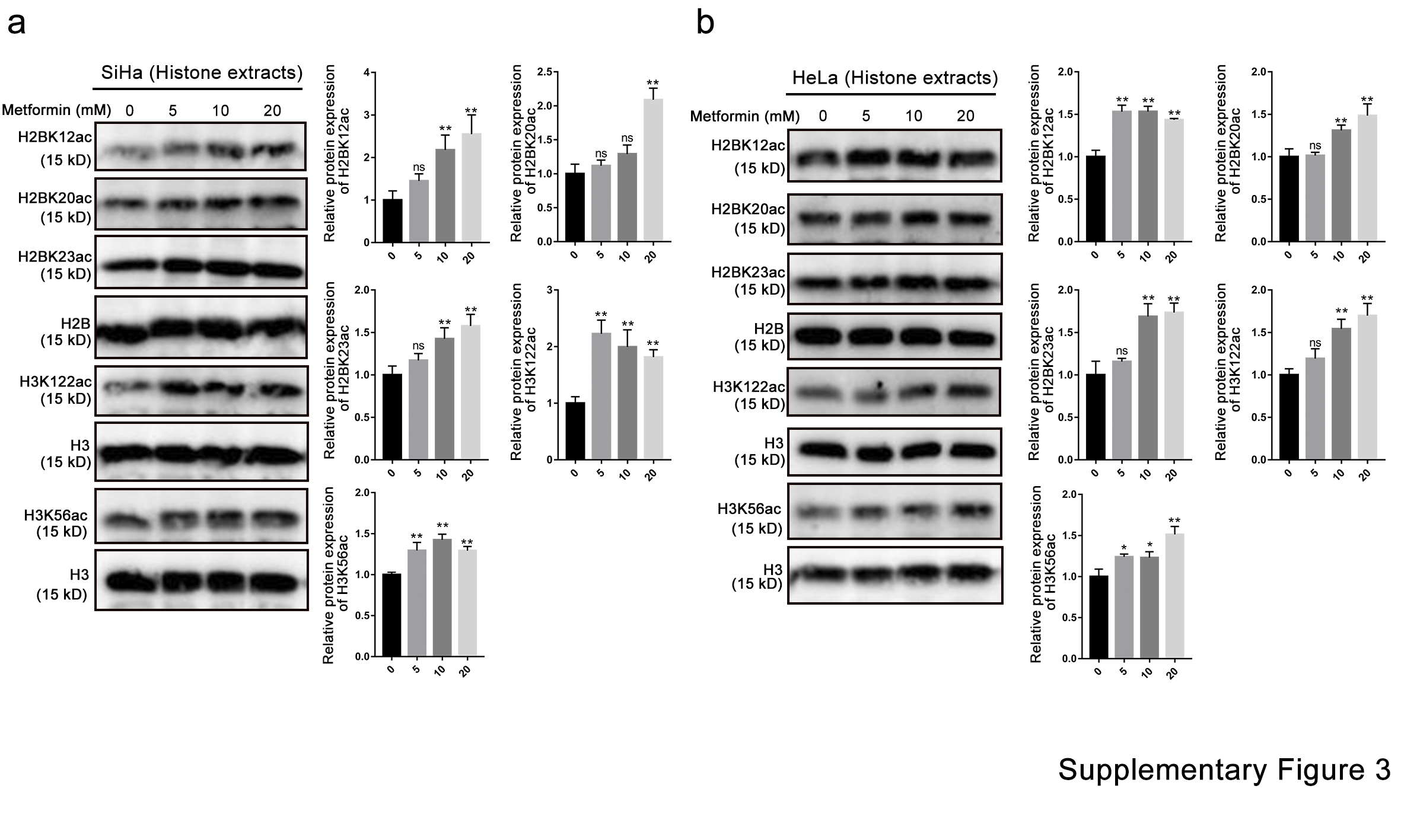

Supplement: Supplementary file 4 — Supplementary Material 4 [file 12964_2024_1687_MOESM4_ESM.tif]

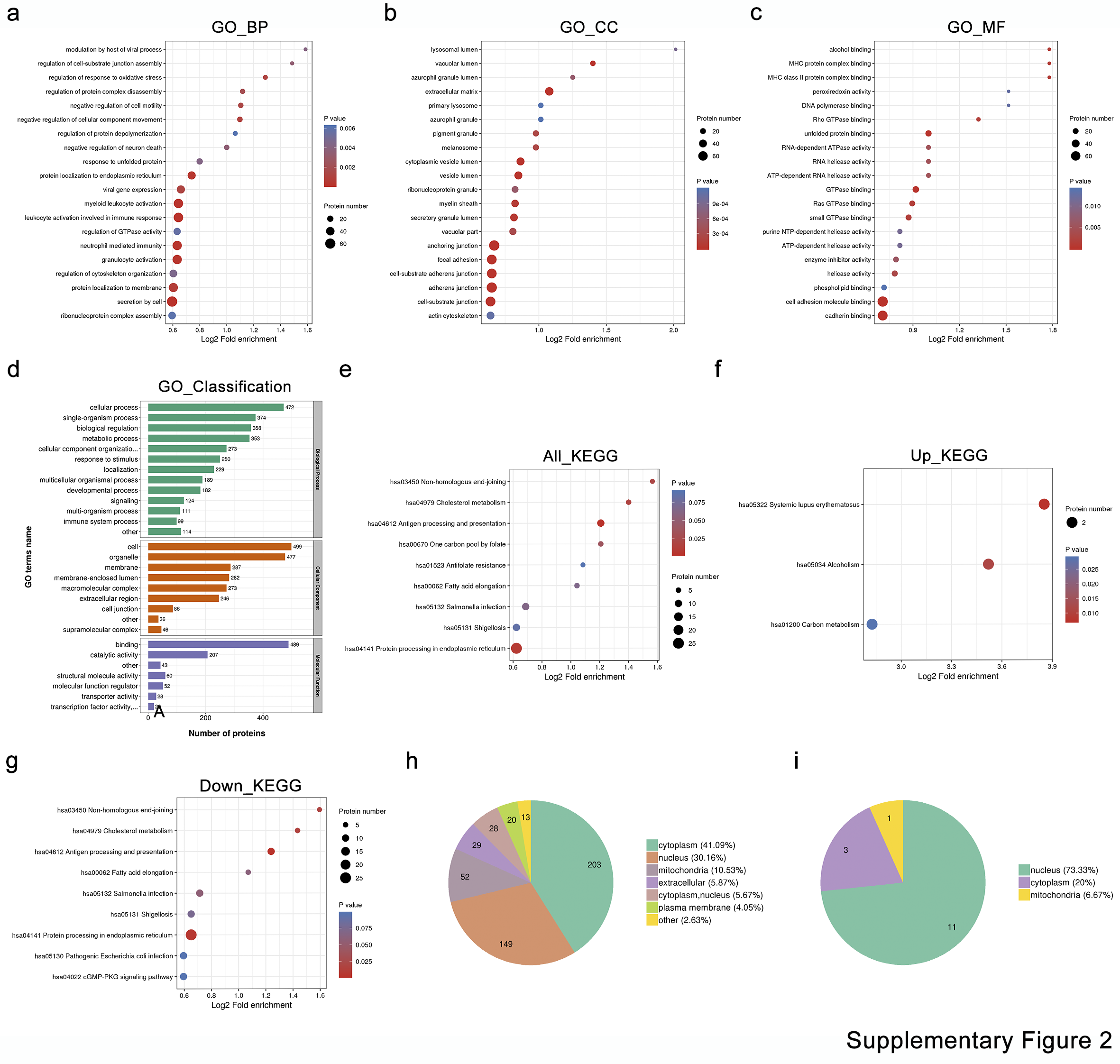

Supplement: Supplementary file 5 — Supplementary Material 5 [file 12964_2024_1687_MOESM5_ESM.tif]

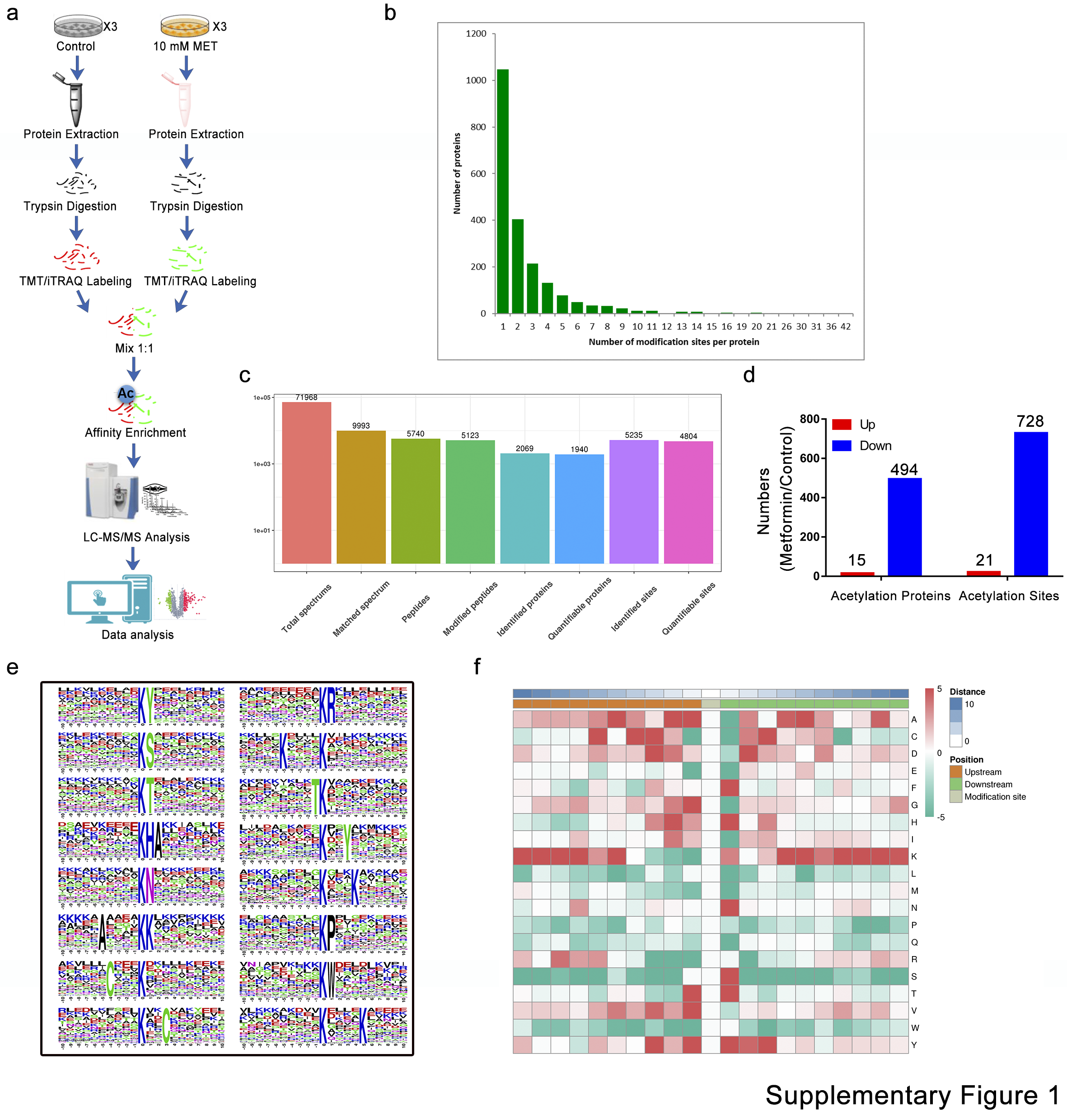

Supplement: Supplementary file 6 — Supplementary Material 6 [file 12964_2024_1687_MOESM6_ESM.tif]
